# Patterns of psychotropic drug prescriptions and general practice consultations among community-dwelling older people with dementia during the first two years of the COVID-19 pandemic

**DOI:** 10.1186/s12877-024-04708-9

**Published:** 2024-02-01

**Authors:** Jiamin Du, Huibert Burger, Thijmen Kupers, Karina Sulim, Maarten T. Homburg, Jean W. M. Muris, Tim C. olde Hartman, Sytse U. Zuidema, Lilian L. Peters, Sarah I. M. Janus

**Affiliations:** 1 Department of Primary and Long-Term Care, University of Groningen, University Medical Centre Groningen, Groningen, the Netherlands; 2 Department of Family Medicine, Maastricht University Medical Centre, CAPHRI Care and Public Health Research Institute, Maastricht, the Netherlands; 3 Department of Primary and Community Care, Radboud University Nijmegen Medical Centre, Nijmegen, the Netherlands; 4 Alzheimer Centre Groningen, Groningen, the Netherlands; 5 Amsterdam University Medical Centre, Vrije Universiteit Amsterdam, Midwifery Science, AVAG, Amsterdam Public Health, Amsterdam, the Netherlands

**Keywords:** Dementia, Psychotropic drugs, General practice, Aged, Independent living, COVID-19

## Abstract

**Background:**

The COVID-19 pandemic and subsequent lockdown measures had serious implications for community-dwelling older people with dementia. While the short-term impacts of the pandemic on this population have been well studied, there is limited research on its long-term impacts. Quantifying the long-term impacts may provide insights into whether healthcare adaptations are needed after the acute phase of the pandemic to balance infection prevention measures with healthcare provision. This study aims to examine patterns of psychotropic drug prescriptions and general practice consultations in community-dwelling older people with dementia during the first two years of the pandemic.

**Methods:**

We utilised routine electronic health records from three Dutch academic general practice research networks located in the North, East, and South, between 2019 and 2021. We (1) compared the weekly prescription rates of five groups of psychotropic drugs and two groups of tracer drugs, and weekly general practice consultation rates per 1000 participants, between the first two years of the pandemic and the pre-pandemic phase, (2) calculated changes in these rates during three lockdowns and two relaxation phases relative to the corresponding weeks in 2019, and (3) employed interrupted time series analyses for the prescription rates. Analyses were performed for each region separately.

**Results:**

The study population sizes in the North, East, and South between 2019 and 2021 were 1726 to 1916, 93 to 117, and 904 to 960, respectively. Data from the East was excluded from the statistical analyses due to the limited sample size. During the first two years of the pandemic, the prescription rates of psychotropic drugs were either lower or similar to those in the pre-pandemic phase, with differences varying from -2.6‰ to -10.2‰. In contrast, consultation rates during the pandemic were higher than in the pre-pandemic phase, increasing by around 38‰.

**Conclusions:**

This study demonstrates a decrease in psychotropic drug prescriptions, but an increase in general practice consultations among community-dwelling older people with dementia during the first two years of the pandemic. However, reasons for the decrease in psychotropic drug prescriptions are unclear due to limited information on the presence of neuropsychiatric symptoms and the appropriateness of prescribing.

**Supplementary Information:**

The online version contains supplementary material available at 10.1186/s12877-024-04708-9.

## Background

During the SARS-CoV-2 virus (COVID-19) pandemic, social distancing, wearing face masks, and lockdown measures were taken to limit the spread of the virus. As a vulnerable group, older people with dementia (OlderPwD) were at a higher risk of infection and death related to COVID–19 [1–4]. Furthermore, they also had a higher risk of breakthrough infections following the vaccination, ranging from 8.6% to 12.4% [5]. Moreover, at the time, OlderPwD who resided in communities suffered greatly from isolation and lockdown policies, which interrupted healthcare and social support. This group in particular relies on informal carers for assistance with daily activities and healthcare services from general practitioners (GPs) and nurses [6–8]. Given the fact that community-dwelling OlderPwD (CD-OlderPwD) heightened vulnerability to infections and reliance on external assistance in daily living and healthcare, the investigation of the acute or long-term impact of the COVID-19 pandemic on this group holds significant interest, related to both the infection burden and potential delays in healthcare provision.

At the beginning of the pandemic in 2020, CD-OlderPwD experienced deterioration of neuropsychiatric symptoms (NPSs) and cognitive functions [7, 9–13]. Their psychotropic drug prescriptions increased in the initial stage of the pandemic possibly due to the deterioration of NPSs and interruption in healthcare services [7, 10]. Medication review organised by general practitioners in England was postponed during the COVID-19 pandemic, which probably led to inappropriate medication prescriptions [14, 15]. Additionally, utilisation of home care and healthcare services was interrupted [16, 17]. In Canada, for example, CD-OlderPwD made 16% to 50% less use of personal care (care relates to daily living) and therapies compared to 2019 [16]. In Germany, the utilisation of GP or internal medicine specialist consultations by older people in March, April, and May 2020 changed with + 15%, -15%, and -5%, respectively, compared to the corresponding months in 2019 [17].

While the short-term consequences (i.e., of the first wave of the pandemic and/or the first relaxation phase) of the COVID-19 pandemic for this population have been well studied, there remains a gap in understanding its prolonged effects across subsequent waves. For example, a study conducted at a later stage of the pandemic reported that, according to population-based data, antipsychotic prescriptions among people with dementia increased in the first month after the COVID-19 outbreak in most study countries compared to the pre-pandemic levels, which persisted until November 2021 [18]. However, no marked change in antidepressant prescriptions was observed during the same period [18].

The long-term impact of the COVID-19 pandemic on psychotropic drug prescriptions and healthcare utilisation within general practice consultations among CD-OlderPwD remains insufficiently explored. Insight into these long-term patterns could help shape healthcare adaptations following the initial phase of a comparable future pandemic, facilitating the optimal balance between infection prevention measures and healthcare provision. Therefore, this study aims to investigate the short- and long-term changes in the patterns of psychotropic drug prescriptions and general practice consultations among CD-OlderPwD during the first two years of the COVID-19 pandemic in the Netherlands.

## Methods

### Study design and data sources

This retrospective cohort study used routine electronic health records (EHRs) collected between 2019 and 2021 by GPs from three academic general practice research networks in the northern, southern, and eastern regions of the Netherlands. The Academic General Practitioner Development Network (Academisch Huisartsen Ontwikkel Netwerk – AHON) at the time of the study comprised 59 affiliated general practices in the northern region [19]. The Research Network Family Medicine (RNFM) comprised 28 affiliated general practices in the southern region [20]. The Family Medicine Network (FaMe-Net) comprised 6 affiliated general practices in the eastern region [21]. Notably, COVID-19 has spread from south to north in the Netherlands. The northern network (AHON) represented a region with a low prevalence at the start of the pandemic, while the southern network (RNFM) represented a high prevalence region, and the eastern network (FaMe-Net) represented an intermediate prevalence region [22]. Therefore, patterns for different regions were studied separately. The extracted clinical data included recorded diagnoses encoded using the International Classification of Primary Care (ICPC-1) codes, types of consultations (e.g. physical, telephone, or digital consultations, home visits, repeat prescription, and others), recorded prescriptions encoded using the Anatomical Therapeutic Chemical (ATC) classification, and the date of each record [23, 24]. The routine EHRs were pseudonymised before extraction.

The FaMe-Net performed one data extraction at the start of 2022, which included data recorded between 2019 and 2021. The AHON and RNFM performed two separate data extractions at the start of 2021 (including data recorded in 2019 and 2020) and at the start of 2022 (including data recorded in 2021), which were later combined into one dataset. Due to the mandatory pseudonymisation process before data extraction, the patient IDs in AHON and RNFM were generated anew in the extraction at the start of 2022. The yearly population data were pre-cleaned based on four rules, the same as the method used in Homburg TM, et al. study [25], 1) exclude people with a deregistration reason but without a date, 2) exclude people with missing birthdate or born before the date ‘01–01-1919’, 3) exclude people who were registered less than one complete quarter of a year, and 4) exclude people without a valid pseudonym (AHON and RNFM) or valid postal code (FaMe-Net).

### Study population

The inclusion criteria for the target population were older people living at home aged 65 years and older, diagnosed with dementia (ICPC code P70), registered with their GP. The exclusion criteria were: a diagnosis of Down’s syndrome (A90.01), schizophrenia (P72), bipolar disorder (P73.02), or affective psychosis (P73). Individuals with both dementia and Down’s syndrome may have a more complicated course, which can make them less representative of the target population. The presence of psychosis could also lead to psychotropic drug prescriptions which may obscure the association being studied. Logical checks were carried out and in cases where violations were found, we excluded the corresponding data points. Namely, the registration date should be no earlier than the birthdate or later than the deregistration date; the earliest date of registered dementia could not be later than the deregistration date and must be at least 30 years later than the birthdate; the earliest year of registered dementia could not be later than the inclusion year.

We defined a dynamic weekly population based on age, the earliest registered date of dementia, and registration and deregistration status. For each weekly population, we counted the number of prescriptions for five groups of psychotropic drugs and two groups of tracer drugs, and the number of general practice consultations in that week.

### Outcomes

The primary outcome was the number of weekly psychotropic drug prescriptions per 1000 CD-OlderPwD further denoted as the prescription rate. There were five groups of psychotropic drugs: antipsychotics (N05A, excluding lithium and prochlorperazine), antidepressants (N06A), anxiolytics (N05B), hypnotics and sedatives (N05C), and anti-dementia drugs (N06D). We also assessed the number of weekly prescriptions for two groups of tracer drugs, opioids (N02A) and statins (C10AA). These two types of tracer drugs were chosen from the essential medicines defined by WHO before the analyses [26]. Opioid prescribing was expected to increase during the COVID-19 pandemic and statin prescribing was not expected to increase [27, 28]. They can help in understanding the reliability of estimated prescribing patterns.

The secondary outcomes encompassed two key measures: the consultation rate, defined as the number of weekly consultations per 1000 CD-OlderPwD, and the distribution of different types of consultations each week. These types include home visits, physical consultations, telephone consultations, digital consultations (such as through email), repeat prescriptions, and any other types.

### The COVID-19 pandemic phases in the Netherlands

For the study period between 2019 and 2021, we identified seven distinct phases: the pre-pandemic phase, three waves of the COVID-19 pandemic, and two relaxation phases. Each of these phases was determined based on the incidence of positive COVID-19 cases and the implementation of restrictive measures (Table 1) [25, 29, 30].

**Table 1:** Phases of COVID-19 pandemic in the Netherlands from 2019–2021: duration and description

7 Phases	Start week (year)	End week (year)	Description of the COVID-19 infections and restrictive measures
Phase 0	Week 1 (2019)	Week 8 (2020)	*The pre-pandemic phase* -Before the outbreak of COVID-19 in the Netherlands
Phase 1	Week 9 (2020)	Week 22 (2020)	*The first wave of the COVID-19 pandemic* -In week 9, the first positive COVID-19 case in the Netherlands was confirmed -In week 11, the cabinet introduced some basic rules and some measures, such as keeping distance, no gatherings with over 100 people, work from home if possible -In week 13 of 2020, the intelligent lockdown was introduced, everyone should stay home as much as possible, a maximum of 3 people for visit, and all gatherings were forbidden
Phase 2	Week 23 (2020)	Week 40 (2020)	*The first relaxation phase* -Decrease in infection rates and relaxation of the measures
Phase 3	Week 41 (2020)	Week 3 (2021)	*The second wave of the COVID-19 pandemic* -In week 42, a partial lockdown was introduced, which was reinforced in week 45 -In week 49, people could get tested without symptoms if they were in close contact with positive cases -In week 51, full lockdown was introduced, which lasted until the end of phase 4
Phase 4	Week 4 (2021)	Week 16 (2021)	*The second wave of the COVID-19 pandemic (alpha-variant)* -In phase 4 alpha variant was the dominant virus, which was more contagious and caused more hospitalizations than previous virus
Phase 5	Week 17 (2021)	Week 43 (2021)	*The second relaxation phase* -Decrease in infection rates and relaxation of the measures
Phase 6	Week 44 (2021)	Week 52 (2021)	*The third wave of the COVID-19 pandemic (delta variant)* -In week 44, the cabinet has implemented additional measures, such as mandating face mask mandatory in more places and requiring a coronavirus entry pass at more locations, to slow down the spread of the virus - In week 50, the lockdown was introduced

### Statistical analysis

**Fig. 1:**
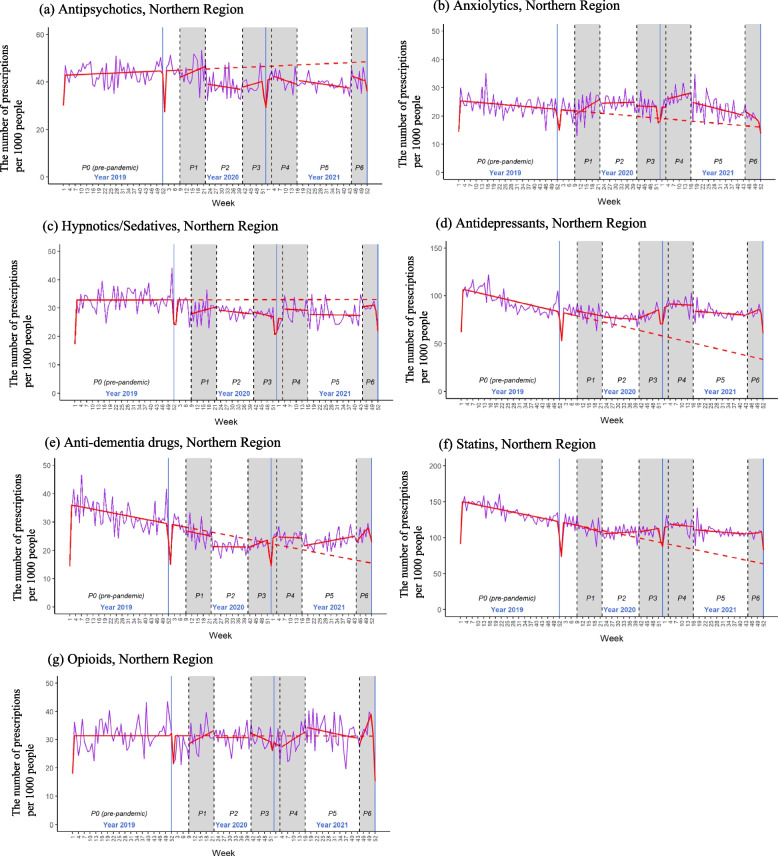
The plots of interrupted time series models of psychotropic and tracer (statins, opioids) drug prescriptions, northern region **a**-**g** P0-P6 refers to phases 0 through 6. The red solid line represents the estimated patterns of psychotropic or tracer drug prescriptions in each phase. The red dashed line shows the counterfactual patterns of psychotropic or tracer drug prescriptions without the COVID-19 impact, derived from the secular trend.The purple line represents the actual patterns of psychotropic or tracer drug prescriptions

To outline the characteristics of the study population we conducted descriptive analysis. Means and standard deviations were calculated for continuous variables, and numbers and percentages for categorical variables. We also reported the number and percentage of CD-OlderPwD who had at least one prescription for psychotropic drugs, opioids, and statins per year. To visualise the data, we created the following figures: (1) a plot of the weekly population from 2019 to 2021, (2) a plot of the weekly consultation rates from 2019 to 2021, which was smoothed using a rolling average of five weeks, and (3) a plot of the weekly percentage of different types of consultations from 2019 to 2021, which was smoothed using the locally estimated scatterplot smoothing regression with a span value of 0.1.

Quantifying the changes in patterns of psychotropic drug prescription rates and consultation rates during the pandemic involved conducting two sets of univariate analyses. The first analysis focused on the first two years of the pandemic as a whole, while the second analysis delved into specific phases within the pandemic. To compare the first two years of the pandemic period with the pre-pandemic phase, we used two types of statistical tests: the unpaired two-sample t-test and the Welch two-sample t-test, which are suitable for data with equal and unequal variances, respectively [31]. For comparing different phases of the pandemic with their corresponding pre-pandemic period, we undertook a two-step approach. First, we calculated the difference in prescription and consultation rates between the pandemic week and the corresponding pre-pandemic week in 2019. Second, we performed one-sample t-tests for each phase (multiple weeks), comparing the observed difference data with a reference value of zero. For not normally distributed data we also performed a Wilcoxon signed rank test. The results of the univariate analyses were reported as differences in means with 95% confidence intervals.

Furthermore, to include the secular trends in the pre-pandemic phase in analyses, we conducted interrupted time-series analyses for the prescription rates for five groups of psychotropic drugs and two groups of tracer drugs. The interrupted time series model estimated the intercept and slope for the pre-pandemic phase, which was used as the reference period. For the six pandemic phases, the changes in intercept and slope were estimated as compared with the pre-pandemic phase 0. We fitted segmented linear regression models. The weekly prescription rate was the dependent variable. The independent variables were: a continuous time variable, i.e. the week number from 2019 to 2021 to assess the baseline trend; six binary variables indicating the start and end of the six pandemic phases to assess the intercept change; six continuous phase-time variables, i.e. the week number in a specific pandemic phase reflecting the interactions between time and the six phases, to assess the slope change; and seven binary variables, i.e. the weeks including the Christmas or New Year holiday, to explicitly fit the number of prescriptions in these weeks. During these holidays, many GPs are not available, which may have led to fewer general practice consultations and prescriptions than usual. These weeks were week 1 and week 52 in 2019; week 1, week 52, and week 53 in 2020; week 51 and week 52 in 2021. We assessed autocorrelation through residual analysis. We reported coefficients with their respective standard errors, R^2^, adjusted R^2^, and F statistic along with its two degrees of freedom. The dashed line represents the counterfactual patterns of psychotropic drug prescriptions, which is derived from the secular trend, indicating what the prescriptions would have been without the influence of the COVID-19 pandemic. In addition, we performed a sensitivity analysis, in which we added a categorical quarter variable to account for a potential seasonality effect.

The statistical analyses were performed in R, version 4.1.1.

## Results

The inclusion process of older people with dementia in three research networks in 2019, 2020, and 2021 is shown separately in Supplementary Table 1. We identified 1858, 1916, and 1726 older people with dementia who were registered in 2019, 2020, and 2021, respectively, in the AHON dataset (northern region); 117, 105, and 93 registered in the FaMe-Net dataset (eastern region); and 921, 904, and 960 registered in the RNFM dataset (southern region). Due to the limited sample size of the Eastern data, we only performed descriptive analyses for the data to ensure the robustness of the results. The study population in the North and the South had similar age distributions (Table 2). The proportion of female older people with dementia stayed stable around 60% in both regions from 2019 to 2021.

**Table 2:** Demographic characteristics and psychotropic drug prescriptions of community-dwelling older people with dementia in the three academic general practice research networks (2019–2021)

	**AHON** **Northern Region**			**FaMe-Net** **Eastern Region**			**RNFM** **Southern Region**		
	**2019**	**2020**	**2021**	**2019**	**2020**	**2021**	**2019**	**2020**	**2021**
Population, N	1858	1916	1726	117	105	93	921	904	960
Age, mean ± SD	83.0 ± 7.2	83.3 ± 7.1	83.2 ± 7.2	82.5 ± 7.7	82.9 ± 7.4	82.6 ± 7.7	83.0 ± 7.6	82.85 ± 7.6	83.0 ± 7.4
Gender, female, N (%)	1138 (61.3)	1181 (61.6)	1041 (60.3)	72 (61.5)	61 (58.1)	51 (54.8)	575 (62.4)	545 (60.3)	589 (61.4)
People who had at least one psychotropic drug prescription, N (%)	820 (44.1)	853 (44.5)	795 (46.1)	39 (33.3)	45 (42.9)	42 (45.2)	383 (41.6)	389 (43.0)	391 (40.7)
Antipsychotics, N (%)	300 (16.2)	314 (16.4)	293 (17.0)	9 (7.7)	15 (14.3)	13 (14.0)	122 (13.3)	139 (15.4)	142 (14.8)
Anxiolytics, N (%)	202 (10.9)	241 (12.6)	211 (12.2)	13 (11.1)	14 (13.3)	14 (15.1)	98 (10.6)	98 (10.8)	103 (10.7)
Hypnotics/Sedatives, N (%)	236 (12.7)	248 (12.9)	225 (13.0)	6 (5.1)	9 (8.6)	6 (6.5)	110 (11.9)	110 (12.2)	101 (10.5)
Antidepressants, N (%)	345 (18.6)	351 (18.3)	331 (19.2)	15 (12.8)	18 (17.1)	18 (19.4)	154 (16.7)	149 (16.5)	158 (16.5)
Anti-dementia drugs, N (%)	207 (11.1)	197 (10.3)	172 (10.0)	12 (10.3)	9 (8.6)	8 (8.6)	86 (9.3)	85 (9.4)	75 (7.8)
People who had at least one opioid prescription, N (%)	305 (16.4)	322 (16.8)	321 (18.6)	16 (13.7)	11 (10.5)	12 (12.9)	128 (13.9)	136 (15.0)	160 (16.7)
People who had at least one statin prescription, N (%)	608 (32.7)	574 (30.0)	513 (29.7)	35 (29.9)	31 (29.5)	36 (38.7)	287 (31.2)	291 (32.2)	330 (34.4)

### The short- and long-term patterns of psychotropic drug prescriptions during the first two years of the COVID-19 pandemic

In the northern region, antidepressants were the most frequently prescribed psychotropic drugs among CD-OlderPwD, with an average prescription rate of 91.9‰ in the pre-pandemic phase, followed by antipsychotics (43.4‰), anti-dementia drugs (31.5‰), hypnotics/sedatives (32.2‰), and anxiolytics (23.3‰) (Table 3). Except for anxiolytic prescriptions, all other psychotropic drug prescriptions showed lower rates during the first two years of the pandemic period than in the pre-pandemic phase, with differences in means ranging from -3.4‰ to -10.2‰ (Table 3). The comparison in psychotropic drug prescription rates between the six pandemic phases and the corresponding pre-pandemic period in 2019 indicated similar patterns. Lower prescription rates were observed for antipsychotics, hypnotics/sedatives, antidepressants, and anti-dementia drugs in most pandemic phases (Table 4 and Supplementary Fig. 5). The one-sample t-test revealed lower prescription rates for anxiolytics, antidepressants, and anti-dementia drugs in the pre-pandemic phase of 2020 (week 1 – week 8) compared to the corresponding weeks in 2019, suggesting a potential decline in these prescription rates in 2019. This finding was further supported by the decreasing secular trends estimated through the interrupted time series analysis (Fig. 1, Supplementary Tables 3 and 5). The residual analysis showed no strong autocorrelation. Compared with the counterfactual patterns, the prescription rates of anxiolytics and antidepressants were higher, and the prescription rate of anti-dementia drugs was lower in phase 2 and started to increase in phase 3 (Fig. 1).

**Table 3:** The weekly prescription and consultation rates per 1000 community-dwelling older people with dementia before and during the pandemic, and the differences

	**The pre-pandemic phase**	**The first two years of the pandemic phase**	
	**Mean (SD)**	**Mean (SD)**	**Difference in means [95%CI]**
**The prescription rate**			
**Northern Region**			
Antipsychotics	43.40 (4.60)^b^	39.95 (4.51)^b^	-3.44 [-4.92, -1.97]^***^
Anxiolytics	23.32 (3.61)	23.39 (4.04)	0.07 [-1.19, 1.33]
Hypnotics/Sedatives	32.23 (4.37)	28.24 (3.23)	-4.00 [-5.20, -2.79]^***^
Antidepressants	91.92 (12.78)	81.68 (7.74)	-10.24 [-13.88, -6.61]^***a^
Anti-dementia drugs	31.53 (5.78)	23.44 (3.11)	-8.09 [-9.70, -6.47]^***a^
Statins	132.14 (15.68)^b^	109.47 (9.81)	-22.67 [-27.16, -18.19]^***a^
Opioids	30.99 (5.15)	31.04 (4.82)	0.05 [-1.56, 1.65]
**Southern Region**			
Antipsychotics	24.28 (4.80)^b^	24.65 (5.13)	0.36 [-1.26, 1.99]
Anxiolytics	27.31 (4.95)	24.08 (4.02)	-3.23 [-4.66, -1.80]^***^
Hypnotics/Sedatives	23.01 (4.38)	22.16 (4.57)	-0.85 [-2.31, 0.61]
Antidepressants	53.58 (8.01)	50.49 (7.09)^b^	-3.09 [-5.51, -0.68]*
Anti-dementia drugs	16.57 (6.12)	13.96 (4.80)^b^	-2.61 [-4.45, -0.76]^**a^
Statins	93.70 (8.02)	95.54 (13.14)	1.83 [-1.50, 5.17]^a^
Opioids	27.54 (5.87)	25.30 (7.26)	-2.25 [-4.44, -0.05]^*^
**The consultation rate**			
Northern region	702.31 (88.84)^b^	740.81 (107.01)	38.50 [7.27, 69.73]^*a^
Southern region	450.07 (66.57)^b^	488.42 (77.34)	38.35 [14.53, 62.18]^**^

*SD* standard deviation, *95% CI* 95% confidence interval

*P* value: ^***^ < 0.001, ^**^ < 0.01, ^*^ < 0.05

^a^The two groups had unequal variance

^b^The group had non-normal distributed data

**Table 4:** The differences in the weekly prescription and consultation rates in 2020 and 2021 compared with the corresponding weeks in 2019

	Phase 0 in 2020 (week 1 – week 8)	Phase 1	Phase 2	Phase 3	Phase 4	Phase 5	Phase 6
	Mean [95% CI]	Mean [95% CI]	Mean [95% CI]	Mean [95% CI]	Mean [95% CI]	Mean [95% CI]	Mean [95% CI]
**Northern Region**							
Antipsychotics	2.26 [-1.49, 6.02]	0.53^a^ [-4.39, 5.44]	-5.09 [-8.07, -2.10]^**^	-4.21 [-7.10, -1.32]^**^	-3.77 [-6.55, -0.98]^*^	-3.56 [-5.47, -1.66]^***^	-3.89 [-8.10, 0.32]
Anxiolytics	-2.77 [-5.29, -0.25]^*^	-1.44 [-4.59, 1.70]	1.58^a^ [-0.19, 3.35]	0.14 [-2.12, 2.40]	1.71 [-1.11, 4.53]	-0.74 [-2.85, 1.38]	-2.83 [-6.44, 0.79]
Hypnotics/Sedatives	-0.60 [-5.31, 4.12]	-2.79^b^ [-5.84, 0.26]	-5.70 [-7.48, -3.93]^***^	-3.99 [-6.30, -1.69]^**^	-3.50 [-5.89, -1.10]^**^	-5.82 [-7.77, -3.87]^***^	-2.97^a^ [-6.87, 0.94]
Antidepressants	-20.00 [-26.18, -13.80]^***^	-21.40 [-27.22, -15.58]^*^	-12.11 [-15.80, -8.41]^***^	-4.79 [-9.69, 0.10]	-16.95 [-20.95, -12.95]^***^	-7.84 [-11.28, -4.39]^***^	-5.43 [-13.08, 2.23]
Anti-dementia drugs	-6.33 [-11.09, -1.58]^*^	-8.51 [-11.96, -5.07]^***^	-9.53 [-11.71, -7.36]^***^	-7.87^a^ [-11.38, -4.35]^***^	-11.28 [-14.30, -8.26]^***^	-7.66 [-9.75, -5.57]^***^	-5.86 [-10.83, -0.88]^*^
Statins	-26.48 [-33.29, -19.68]^***^	-32.07 [-41.17, -22.98]^***^	-24.42 [-29.47, -19.38]^***^	-15.50 [-22.21, -8.79]^***^	-29.44 [-35.06, -23.82]^***^	-25.16 ^a^ [-30.51, -19.81]^***^	-20.52 [-25.96, -15.08]^***^
Opioids	-2.14 [-7.59, 3.32]	-0.73 [-4.24, 2.77]	-1.27 [-3.82, 1.28]	-2.11 [-5.55, 1.32]	0.26 [-3.22, 3.74]	0.37 [-1.77, 2.50]	-2.85 [-8.18, 2.48]
**Southern Region**							
Antipsychotics	8.16 [1.58, 14.73]^*^	5.63 [2.00, 9.26]^**^	-1.36 [-3.57, 0.85]	-0.73 [-5.09, 3.63]	1.84 [-3.62, 7.31]	-0.49 [-3.20, 2.21]	-1.55 [-5.59, 2.49]
Anxiolytics	0.29 [-5.87, 6.45]	-1.85 [-5.84, 2.15]	-7.79 [-10.89, -4.70]^***^	-2.78 [-7.24, 1.67]	-0.63 [-2.83, 1.57]	-4.52 [-6.50, -2.53]^***^	-5.00 [-8.74, -1.25]^*^
Hypnotics/Sedatives	-0.53 [-5.58, 4.52]	-0.61 [-3.73, 2.51]	-4.47 [-6.95, -1.98]^**^	0.76 [-2.54, 4.06]	-3.35 [-6.76, 0.06]	-0.04 [-2.59, 2.51]	-1.60 [-6.52, 3.32]
Antidepressants	-0.78 [-10.24, 8.68]	-1.69^a^ [-6.93, 3.55]	-5.37 [-10.01, -0.73]^*^	-6.96 [-12.23, -1.69]^*^	0.97 [-6.43, 8.37]	-2.23 [-6.47, 2.01]	-12.01 [-16.32, -7.69]^***^
Anti-dementia drugs	0.35^a^ [-6.00, 6.70]	-1.72 [-5.44, 2.01]	-2.53 [-4.31, -0.76]^**^	-3.49 [-6.67, -0.31]^*^	-4.19 [-7.97, -0.40]^*^	-2.36 [-6.13, 1.40]	-4.75 [-11.10, 1.59]
Statins	-2.59 [-12.98, 7.79]	-0.36 [-5.54, 4.81]	-4.30 [-8.88, 0.28]	-12.89^a^ [-23.73, -2.04]^*^	9.18 [0.99, 17.37]^*^	13.38 [9.78, 16.97]^***^	-4.23 [-10.23, 1.77]
Opioids	-5.20 [-14.65, 4.25]	-3.86 [-9.02, 1.30]	-9.61 [-12.83, -6.40]^***^	-3.50^a^ [-8.80, 1.80]	-0.31 [-4.47, 3.85]	-1.00 [-4.19, 2.19]	1.55 [-2.30, 5.39]
**Consultations**							
Northern region	3.89 [-52.88, 60.66]	-82.78 [-144.31, -21.25]^*^	2.50 [-39.93, 44.93]	69.00^a^ [5.46, 132.54]^*^	125.27 [86.02, 164.52]^***^	46.33 [6.35, 86.32]^*^	92.05^a^ [57.63, 126.46]^***^
Southern region	13.47 [ -30.01,56.95]	-10.07 [-61.77, 41.63]	-12.32 [-40.74, 16.10]	13.12 [-47.90, 74.14]	90.29 [20.70, 159.87]^*^	72.67 [46.32, 99.01]^***^	88.32 [36.86, 139.78]^**^

*95% CI* 95% confidence interval

*P* value: ^***^ < 0.001, ^**^ < 0.01, ^*^ < 0.05

^a^The data is not normally distributed. The Wilcoxon signed rank test showed similar statistical results to the one-sample t-test

^b^The data is not normally distributed. The Wilcoxon signed rank test showed different statistical result, which was statistic significant, with *p* value = 0.04

**Fig. 2:**
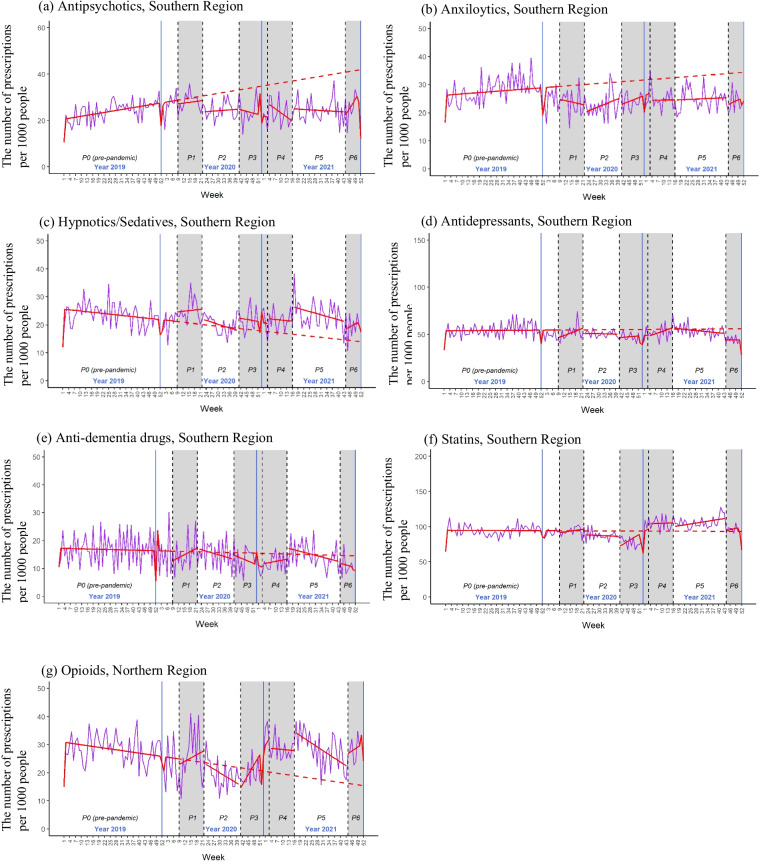
The plots of interrupted time series models of psychotropic and tracer (statins, opioids) drug prescriptions, southern region **a**-**g** P0-P6 refers to phases 0 through 6. The red solid line represents the estimated patterns of psychotropic or tracer drug prescriptions in each phase. The red dashed line shows the counterfactual patterns of psychotropic or tracer drug prescriptions without the COVID-19 impact, derived from the secular trend. The purple line represents the actual patterns of psychotropic or tracer drug prescriptions

In the southern region, antidepressants were also the most frequently prescribed psychotropic drug in CD-OlderPwD, with an average prescription rate of 53.6‰ in the pre-pandemic phase, followed by anxiolytics (27.3‰), antipsychotics (24.3‰), hypnotics/sedatives (23.0‰), and anti-dementia drugs (16.6‰) (Table 3). A decline in prescription rates was observed for anxiolytics, antidepressants, and anti-dementia drugs during the first two years of the pandemic period (Table 3). Additionally, the one-sample t-test showed that the weekly prescription rates for psychotropic drugs decreased in at least one of the six pandemic phases, with the exception of antipsychotics (Table 4). The rate of antipsychotic prescription was higher in pre-pandemic phase in 2020 and in the first wave of COVID-19 pandemic than in corresponding weeks in 2019. Similarly, an increasing secular trend in the prescription rate of antipsychotics was found in the pre-pandemic phase (Fig. 2 and Supplementary Table 4). Antipsychotic prescription rates in pandemic phases were lower compared with its counterfactual patterns. Hypnotics/Sedatives showed a decreasing secular trend in the pre-pandemic phase. Its prescription rates during the pandemic phases were higher compared with the counterfactual patterns, although not statistically significant.

The prescription rate of statins (i.e. one of the tracer drugs) in both the first two years of the pandemic period and six pandemic phases were lower than in the pre-pandemic phase in the northern region. However, the result of the interrupted time series analysis showed a secular downtrend of statin prescription rate in the pre-pandemic phase in the North. Compared with the counterfactual patterns, the prescription rate is higher during the pandemic phases. In the southern region, the prescription rates of statins were similar between pandemic and pre-pandemic phases. For opioids, the other tracer drugs, the prescription rates in the North either during the first two years of the pandemic period or in six pandemic phases were similar to that of the pre-pandemic phase. While the southern region had lower opioid prescription rates during the first two years of the pandemic period, especially in the first relaxation phase. There was a secular downtrend of opioid prescription rate in the South, with an increase in the slope of the second wave of the pandemic (phase 3) and a decrease in the slope of the second relaxation phase (phase 5) (Supplementary Table 4).

The sensitivity analyses revealed seasonal trends in prescription rates for antidepressants and opioids in both regions (Supplementary Tables 5 and 6), but they were variable and not robust. Additionally, the anxiolytic prescription rate in the South also showed a seasonal trend with an increase in the third and the fourth quarters. The patterns of prescription rates for psychotropic drugs, statins, and opioids were similar to the results of original interrupted time series analyses, after accounting for seasonality.

### The short- and long-term patterns of general practice consultations during the first two years of the COVID-19 pandemic

The general practice consultation rates in CD-OlderPwD in the North and South were 38.5‰ and 38.4‰ higher during the first two years of the pandemic period than those observed in the pre-pandemic phase, respectively (Table 3). Compared with the corresponding period in 2019, the weekly general practice consultation rates in the North showed a decrease of 82.8‰ in the first wave of the pandemic (phase 1), a similar level in the first relaxation phase (phase 2), and increases in the following phases (phases 3 to 6) (Table 4 and Supplementary Fig. 4). While the weekly general practice consultation rates in the South were at similar a level in phase 1, and at higher levels from phase 4 onwards.

The percentage of different types of consultations differed per research network in the pre-pandemic phase (Supplementary Table 2 and Supplementary Fig. 2). The distribution of consultation types in the pre-pandemic phase was relatively more balanced in the North, with 27.0% of physical consultations, 20.6% of telephone consultations, 13.5% of home visits, and other types of consultations. However, in the South, physical consultations were around half, telephone consultations 5.5%, and home visits 14.4%. Despite the between regional differences in the pre-pandemic phase, the change in the percentage of different types of consultations showed similar patterns during the first two years of the pandemic. The percentage of home visits first showed a deep decline after the outbreak of COVID-19, then increased gradually several weeks later, and remained at a slightly lower level than in the pre-pandemic phase. Simultaneously, the percentage of telephone consultations showed contrast trends, increased firstly and then decreased, and remained at a higher level.

## Discussion

### Summary of findings

During the COVID-19 pandemic, prescription of psychotropic drugs among CD-OlderPwD decreased, but the number of general practice consultations increased. Compared with the corresponding period in 2019, prescriptions for most psychotropic drugs in the North and South were similar during the first wave of the pandemic (phase 1), declined in the first relaxation phase (phase 2), and stabilized at similar or lower levels in subsequent phases (phases 3 to 6). The number of general practice consultations in the North initially decreased, but eventually rose to pre-pandemic levels towards the end of phase 1. In contrast, the number of general practice consultations in the South remained stable during this phase. Since the beginning of the second wave of the pandemic, the number of GP consultations has increased in both regions.

### The short- and long-term patterns of psychotropic drug prescriptions

Contrary to our findings, previous studies reported that CD-OlderPwD used more antipsychotics and antidepressants in the early stage of the pandemic [7, 10, 18]. One possible reason could be that CD-OlderPwD in our study might experience fewer NPSs during the pandemic because of their smaller social circles, resulting in fewer triggers for NPSs and an increased sense of safety [32]. However, it is also possible that CD-OlderPwD experienced an increase in NPSs, but they had delayed healthcare-seeking behaviours, or their GPs did not recognise the symptom, or did not consider it a sufficient reason to prescribe psychotropic drugs, or delayed treatments [14, 33, 34]. Although GPs had switched to telephone consultations, older people who were unable to use telephone consultations independently and did not receive sufficient support may have been overlooked. Telephone consultations are reported to not work as effective as face-to-face consultations, so some NPSs might not be detected and the real need for the treatment of NPSs, such as psychotropic drugs, may have been higher [35]. Alternatively, the Netherlands took measures quickly in April 2020 to support informal caregivers, such as the Informal Care Line providing tips and advice, daily support from volunteers, municipality, district nurses, and GPs [36]. This could be another reason why CD-OlderPwD in the Netherlands did not have more psychotropic drug prescriptions. We did not see a rebound in psychotropic drug prescribing after the termination of the first lockdown, but rather a reduction in prescriptions. One possible reason could be that NPSs are fluctuating and transient, so compensatory treatment may not be necessary. Additionally, both GPs and the public gradually adapted to the new routine. Older people with dementia and their caregivers may have developed strategies to cope with pandemic-related stress, potentially resulting in reduced NPSs and, consequently, fewer psychotropic drug prescriptions compared to the first wave. Our study revealed that during the first relaxation phase, the rate of general practice consultations returned to the 2019 level. Increased access to general practice consultations might also contribute to a decrease in NPSs or fewer psychotropic drug prescriptions.

Since the second wave of the pandemic, psychotropic drug prescription rates were either similar or lower than those in the corresponding pre-pandemic period. For psychotropic drugs that did not show a decreasing secular trend in the pre-pandemic phase, similar or lower long-term patterns were also revealed by time series analyses. It is reported that during the first two years of the pandemic period from 2020 to 2021, there was an increase in antipsychotic prescriptions in most study countries (decreases were found as well) and stability in antidepressant prescriptions [18]. Although we could not rule out the possibility that the lower psychotropic drug prescription rates observed in this study were caused by the interruption in health care services [14, 33, 34], the decreases are more likely to be affected by other factors, especially in the long run, which was supported by the increase in general practice consultation rates from the second wave of the pandemic onwards. Besides, we suppose the dynamic population tended to be healthier or had less severe dementia as time passed by, since older people with dementia had a high infection and mortality risk of the COVID-19 virus, and people with newly diagnosed dementia joined the dynamic weekly population [1–4]. Alternatively, restrictions during the pandemic may be a protective factor for these groups of the population. They might experience fewer triggers that caused NPSs and feel safe and less stress due to a smaller social circle [32]. Moreover, one study compared the changes in mental health among individuals with dementia between the pandemic group (assessed before and during the pandemic) and the pre-pandemic group (evaluated twice before the pandemic), and concluded that the pandemic had little to no effect on their mental health [37].

Opioids were somewhat stable during the pandemic phases in both regions. But the prescription rate of statins had a decreasing secular trend in 2019 in the North. While the prescription rate of statins in the South remained stable. However, we have no explanation for the decreasing secular trend in statins in the North during the pre-pandemic phase. Besides, the limited data points in the pre-pandemic phase, only 52 weeks in 2019, might cause unrobust patterns in this period.

### The short- and long-term patterns of general practice consultations

This study showed that the consultation patterns of people with dementia with GPs differed between the North and the South during the first wave of the pandemic (phase 1). This might have been influenced by the spread of COVID-19 in the Netherlands, namely from South to North [22]. At the beginning of the first wave of the pandemic, there was no infected case in the North yet. The drop in the number of consultations in the North at the beginning may have been influenced by the lockdown policy and the fear of getting infected [35]. The decrease in general practice consultations and healthcare services in the early stage of the pandemic were also reported by previous studies [16, 17]. However, consultation rates in the North started to increase several weeks later. The need for GP consultations might have prevailed over the desire to avoid them in order to reduce infection risk. Delays in healthcare could also have played a role in the resumption of GP consultations. Older people with dementia in the South were affected by both COVID-19 infections and the lockdown policy at the beginning of the pandemic. As a vulnerable group, they were at high risk of infections and severe symptoms, which could be a reason for the stable pattern in their consultation rates [1–4].

During the second wave of the pandemic and the subsequent phases, both regions had higher consultation rates. These increases in consultations could be related to flu and COVID-19 vaccinations. In the Netherlands, seniors aged 60 and above who live at home are eligible for free flu vaccination from their own GP in October or November every year [38]. It was expected that more older people would be willing to take flu vaccinations to protect themselves during the pandemic. The COVID-19 vaccination injection started with the oldest (over 90) in late January 2021 [39, 40]. Older people with dementia might get COVID-19 vaccinations from their GP if they could not travel to a Municipal Public Health Services vaccination location [40]. All people aged over 60 were vaccinated by the end of April 2021 [41]. The repeat injection in June and booster vaccinations in November were prioritized based on age, starting with the oldest individuals [42, 43]. Another possible reason for the high number of consultations could be complaints and symptoms due to COVID-19 infections, as older people with dementia vaccinated or unvaccinated, are at higher risk of infections than other populations [2, 5]. Older people with dementia had high demand for care during the pandemic, both in the short and long term.

Looking at the percentage of different types of consultations, we see similar patterns in both regions. During the first lockdown, home visits were interrupted the most. Meanwhile, GPs quickly adapted their consultation strategy and switched to telephone consultations. During subsequent pandemic phases, telephone consultations remained at a slightly higher level than in the pre-pandemic phases. Similar patterns in shifts of different types of consultations were found in a broader population [44, 45]. It was hypothesized that older people with dementia have a degree of resilience towards remote telephone consultations, independently or with the assistance of caregivers [34, 35]. However, physical consultations remained the most commonly used type in both regions, which were also reported to be preferred by caregivers and GPs [35, 46].

### Strengths and limitations

Previous studies focusing on CD-OlderPwD were conducted in 2020, mostly the first wave of the pandemic. Since the pandemic lasted for a longer period, this study explored the patterns of psychotropic drug prescriptions and general practice consultations during a two-year period, which included three waves of the pandemic. The prescriptions and consultations in different pandemic waves and relaxation periods were compared with the corresponding periods in 2019. In this way, we could study both the short-term and long-term impacts of the pandemic. Studying general practice consultations, reflecting the use of primary health care during the pandemic, helped to interpret whether the change in psychotropic drug prescriptions was due to interrupted healthcare services.

However, there were several limitations of this study. For the pre-pandemic phase, we only had data from 2019, which made it difficult to predict robust secular trends. The points in some phases were limited, so the fitted segmented linear regression models were less robust. Thus, readers should be cautious when interpreting the results of time series analyses. The raw number of weekly psychotropic drug prescriptions were small at some time points, varying from 20 to 188 in the North and from 4 to 55 in the South. Future studies could include more people to increase the number to get more robust results. Finally, we had no information on the incidence of neuropsychiatric symptoms, making it difficult to assess the appropriateness of psychotropic medication prescriptions.

## Conclusions

This study assessed the patterns of psychotropic drug prescriptions and general practice consultations in CD-OlderPwD during the first two years of the COVID-19 pandemic. In the first wave of the pandemic, psychotropic drug prescriptions remained at similar levels, but general practice consultations were interrupted. In subsequent phases, we saw a decrease in psychotropic drug prescriptions and an increase in general practice consultations. These findings suggest that the short-term and long-term impact of the pandemic differed. While we have no clear explanation for the decrease in psychotropic drug prescriptions, it is more likely due to fewer NPSs, delays in seeking healthcare and/or providing treatments related to NPSs, rather than interruptions in general practice consultations. Future research on the long-term patterns of psychotropic drug prescriptions during a pandemic might consider adding information on neuropsychiatric symptoms and/or the prescription appropriateness, which would make the results instructive for general practice. Furthermore, our findings indicate that policymakers and healthcare professionals should closely monitor neuropsychiatric symptoms experienced by CD-OlderPwD during future pandemics to optimize both patient care and infection control.

### Supplementary Information

**Additional file 1: Table S1.** Flow chart for the recruitment of the study population in different research networks each year. **Table S2.** The number of prescriptions for psychotropic drugs and two tracer drugs, and general practice consultations of community-dwelling older people with dementia in different research networks by year (2019-2021). **Table S3.** The interrupted time-series model of the rate of prescription for psychotropic drugs and two tracer drugs in community-dwelling older people with dementia in different phases of the COVID-19 pandemic in the northern region of the Netherlands. **Table S4.** The interrupted time-series model of the rate of prescription for psychotropic drugs and two tracer drugs in community-dwelling older people with dementia in different phases of the COVID-19 pandemic in the southern region of the Netherlands. **Table S5.** The interrupted time-series model of the rate of prescription for psychotropic drugs and two tracer drugs in community-dwelling older people with dementia in different phases of the COVID-19 pandemic in the northern region of the Netherlands, adjusted for quarter seasonality. **Table S6.** The interrupted time-series model of the rate of prescription for psychotropic drugs and two tracer drugs in community-dwelling older people with dementia in different phases of the COVID-19 pandemic in the southern region of the Netherlands, adjusted for quarter seasonality. **Figure S1.** The number of weekly study population in the northern and southern regions from 2019 to 2021. **Figure S2.** The percentage of different types of consultations per week from 2019 to 2021. **Figure S3.** The rate of weekly general practice consultations per 1000 community-dwelling older people with dementia from 2019 to 2021. **Figure S4.** The absolute change in the rate of weekly general practice consultations per 1000 community-dwelling older people with dementia, compared with corresponding weeks in 2019. **Figure S5.** The absolute change in the rate of weekly prescriptions per 1000 community-dwelling older people with dementia, compared with corresponding weeks in 2019.

## Data Availability

The data that support the findings of this study are routine electronic health records from three academic general practice research networks, namely, the Academic General Practitioner Development Network (AHON), the Research Network Family Medicine (RNFM), and the Family Medicine Network (FaMe-Net). The data set is not freely available: permission must be sought along with potential associated fees, before access is granted. These arrangements are in place to ensure proper data usage. More information and inquiries about the harmonised linked datasets can be directed to ahon@umcg.nl.

## Abbreviations

OlderPwD: Older people with dementia
CD-OlderPwD: Community-dwelling older people with dementia
GPs: General practitioners
NPSs: Neuropsychiatric symptoms
EHRs: Electronic health records
AHON: The Academic General Practitioner Development Network (Academisch Huisartsen Ontwikkel Netwerk)
RNFM: The Research Network Family Medicine
FaMe-Net: The Family Medicine Network
ICPC: The International Classification of Primary Care codes
ATC: The Anatomical Therapeutic Chemical classification
