# Supplementary material for: Patterns of psychotropic drug prescriptions and general practice consultations among community-dwelling older people with dementia during the first two years of the COVID-19 pandemic
Source: BMC Geriatr. 2024 Feb 1;24:120. doi: 10.1186/s12877-024-04708-9 (PMC10832125; doi:10.1186/s12877-024-04708-9)

**Supplementary Materials**

Table S1. Flow chart for the recruitment of the study population in different research networks each year.

|  | | **AHON**  **Northern Region** | | | **FaMe-Net**  **Eastern Region** | | | **RNFM**  **Southern Region** | | |
| --- | --- | --- | --- | --- | --- | --- | --- | --- | --- | --- |
|  | | **2019** | **2020** | **2021** | **2019** | **2020** | **2021** | **2019** | **2020** | **2021** |
| All registered population, N | | 261789 | 267219 | 265084 | 41100 | 42015 | 40591 | 122750 | 124106 | 129197 |
| People with dementia, N | | 2547 | 2286 | 1825 | 228 | 186 | 142 | 1198 | 969 | 1024 |
| People aged 65 years old each year, N | | 2467 | 2212 | 1770 | 222 | 180 | 138 | 1141 | 928 | 979 |
| Exclusion  criteria | Down Syndrome, N | 3 | 2 | 1 | 0 | 0 | 0 | 1 | 1 | 2 |
|  | Schizophrenia, N | 3 | 3 | 3 | 2 | 2 | 1 | 3 | 2 | 1 |
|  | Bipolar disorder, N | 11 | 10 | 7 | 0 | 0 | 0 | 5 | 5 | 6 |
|  | Affective psychosis, N | 12 (1)^a^ | 10 (1)^a^ | 8 | 1 | 1 | 1 | 7 (1)^a^ | 3 (1)^a^ | 3 |
|  | deregistration date before (<=) Registration date after, N | 0 | 0 | 0 | 0 | 0 | 0 | 0 | 0 | 0 |
|  | The registration date before (<) the birthdate, N | 29 | 26 | 25 | 63 | 49 | 43 | 13 | 11 | 7 |
|  | The earliest registered dementia date after (>=) the deregistration date, N | 1 | 1 | 0 | 0 | 0 | 0 | 0 | 0 | 0 |
|  | The earliest registered dementia date before (< )30 + the birthdate, N | 0 | 0 | 0 | 0 | 0 | 0 | 0 | 0 | 0 |
|  | the earliest registered dementia year after (>) the inclusion year, N | 551 | 245 | 0 | 39 | 23 | 0 | 192 | 3 | 0 |
| Final population, N | | 1858 | 1916 | 1726 | 117 | 105 | 93 | 921 | 904 | 960 |

^a:^ There was one patient who had both diagnoses of bipolar disorders and affective psychosis.

Table S2. The number of prescriptions for psychotropic drugs and two tracer drugs, and general practice consultations of community-dwelling older people with dementia in different research networks by year (2019-2021).

|  | | | **AHON**  **Northern** **Region** | | | **FaMe-Net**  **Eastern Region** | | | **RNFM**  **Southern Region** | | |
| --- | --- | --- | --- | --- | --- | --- | --- | --- | --- | --- | --- |
|  | | | **2019** | **2020** | **2021** | **2019** | **2020** | **2021** | **2019** | **2020** | **2021** |
| Psychotropic drug prescriptions, N | | | 19568 | 18322 | 16371 | 511 | 591 | 383 | 6449 | 5791 | 6142 |
|  | | Antipsychotics, N | 3984 | 3946 | 3405 | 99 | 90 | 45 | 1087 | 1073 | 1024 |
|  | | Anxiolytics, N | 2002 | 2110 | 1861 | 137 | 175 | 99 | 1214 | 972 | 1024 |
|  | | Hypnotics/Sedatives, N | 2749 | 2572 | 2261 | 64 | 38 | 24 | 1021 | 967 | 1036 |
|  | | Antidepressants, N | 8070 | 7443 | 6924 | 153 | 239 | 171 | 2379 | 2150 | 2478 |
|  | | Anti-dementia drugs, N | 2763 | 2251 | 1920 | 58 | 49 | 44 | 748 | 629 | 580 |
| Opioids, N | | | 2943 | 2974 | 2843 | 88 | 49 | 29 | 1285 | 984 | 1366 |
| Statins, N | | | 11534 | 9878 | 8851 | 151 | 267 | 324 | 4075 | 3443 | 4374 |
| Consultations, N | | | 57162 | 58822 | 59265 | 2394 | 2312 | 2560 | 17279 | 16754 | 20730 |
|  | Physical consultations, N (%) | | 15423 (26.98) | 15078 (25.63) | 14407 (24.31) | 580 (24.23) | 415 (17.95) | 411 (16.05) | 8250 (47.75) | 8624 (51.47) | 8767 (43.29) |
|  | Phone consultations, N (%) | | 11782 (20.61) | 14675 (24.95) | 14358 (24.23) | 750 (31.3) | 1051 (45.46) | 853 (33.32) | 942 (5.45) | 1546 (9.23) | 1863 (8.99) |
|  | Digital consultations, N (%) | | 59 (0.10) | 105 (0.18) | 97 (0.16) | 33 (1.38) | 45 (1.95) | 28 (1.09) | 26 (0.15) | 52 (0.31) | 109 (0.53) |
|  | Home visits, N (%) | | 7693 (13.46) | 6119 (10.40) | 6615 (11.16) | 437 (18.25) | 330 (14.27) | 261 (10.20) | 2479 (14.35) | 1913 (11.42) | 2305 (11.12) |
|  | Repeat prescriptions, N (%) | | 4604 (8.05) | 4626 (7.86) | 3920 (6.61) | 279 (11.65) | 216 (9.34) | 738 (28.83) | 2099 (12.15) | 1737 (10.37) | 2528 (12.19) |
|  | Other types, N (%) | | 17601 (30.79) | 18219 (30.97) | 19868 (33.52) | 315 (13.16) | 255 (11.02) | 269 (10.51) | 3483 (20.16) | 2882 (17.2) | 5158 (24.88) |

Table S3. The interrupted time-series model of the rate of prescription for psychotropic drugs and two tracer drugs in community-dwelling older people with dementia in different phases of the COVID-19 pandemic in the northern region of the Netherlands

| **AHON**  **Northern Region** | | **Antipsychotics** | **Anxiolytics** | **Hypnotics/Sedatives** | **Antidepressants** | **Anti-dementia Drugs** | **Statins** | **Opioids** |
| --- | --- | --- | --- | --- | --- | --- | --- | --- |
| Pre-pandemic | Intercept (SE) | 42.80 (1.07)*** | 25.51 (0.91)*** | 32.74 (0.90)*** | 107.74 (1.85)*** | 36.27 (0.91)*** | 151.25 (2.29)*** | 31.39 (1.26)*** |
|  | Time (SE) | 0.04 (0.03) | -0.06 (0.03)* | 0.00 (0.03) | -0.47 (0.05)*** | -0.13 (0.03)*** | -0.56 (0.07)*** | -0.00 (0.04) |
| Phase 1 | Intercept change (SE) | -3.36 (2.45) | -1.80 (2.09) | -5.05 (2.07)* | 5.20 (4.26) | -1.30 (2.10) | 0.67 (5.25) | -3.20 (2.89) |
|  | Slope change (SE) | 0.32 (0.26) | 0.48 (0.22)* | 0.19 (0.22) | 0.06 (0.45) | -0.01 (0.22) | -0.24 (0.56) | 0.37 (0.31) |
| Phase 2 | Intercept change (SE) | -6.22 (2.41)* | 3.45 (2.06) | -3.51 (2.03) | 5.00 (4.19) | -5.16 (2.06)* | -4.66 (5.16) | -0.57 (2.84) |
|  | Slope change (SE) | -0.16 (0.18) | 0.08 (0.15) | -0.08 (0.15) | 0.34 (0.31) | 0.12 (0.15) | 0.72 (0.39) | -0.01 (0.21) |
| Phase 3 | Intercept change (SE) | -8.49 (2.86)** | 3.65 (2.44) | -4.13 (2.42) | 11.50 (4.97)* | -3.43 (2.45) | 7.45 (6.13) | 1.29 (3.37) |
|  | Slope change (SE) | 0.20 (0.23) | 0.03 (0.19) | -0.17 (0.19) | 1.34 (0.39)*** | 0.40 (0.19)* | 1.06 (0.49)* | -0.30 (0.27) |
| Phase 4 | Intercept change (SE) | -4.01 (3.36) | 6.93 (2.87)* | -3.26 (2.84) | 34.91 (5.85)*** | 2.79 (2.88) | 27.92 (7.21)*** | -4.34 (3.97) |
|  | Slope change (SE) | -0.32 (0.29) | 0.23 (0.25) | -0.04 (0.25) | 0.36 (0.51) | 0.10 (0.25) | 0.28 (0.63) | 0.45 (0.34) |
| Phase 5 | Intercept change (SE) | -6.43 (3.24)* | 6.75 (2.77)* | -5.24 (2.74) | 33.50 (5.63)*** | 1.24 (2.78) | 27.36 (6.95)*** | 3.18 (3.82) |
|  | Slope change (SE) | -0.16 (0.10) | -0.11 (0.09) | -0.01 (0.09) | 0.31 (0.18) | 0.26 (0.09)** | 0.33 (0.22) | -0.15 (0.12) |
| Phase 6 | Intercept change (SE) | -5.35 (4.94) | 5.60 (4.22) | -2.64 (4.17) | 41.95 (8.59)*** | 5.48 (4.23) | 36.17 (10.59)*** | -4.78 (5.83) |
|  | Slope change (SE) | -0.33 (0.74) | -0.42 (0.63) | 0.10 (0.63) | 1.32 (1.29) | 0.96 (0.64) | 0.85 (1.59) | 1.80 (0.87)* |
| Week 1 in 2019 | | -12.73 (4.05)** | -11.11 (3.46)** | -15.53 (3.42)*** | -45.62 (7.04)*** | -21.80 (3.47)*** | -59.65 (8.69)*** | -13.47 (4.78)** |
| Week 52 in 2019 | | -1.14 (4.01) | -3.10 (3.42) | -8.54 (3.39)* | -7.84 (6.97) | -6.37 (3.44) | -20.87 (5.59)* | 1.02 (4.73) |
| Week 1 in 2020 | | -17.32 (4.01)*** | -7.31 (3.43)* | -8.66 (3.39)* | -29.79 (6.98)*** | -14.25 (3.44)*** | -47.33 (8.60)*** | -9.80 (4.73)* |
| Week 52 in 2020 | | -7.15 (4.15) | -5.63 (3.55) | -6.11 (3.51) | -15.69 (7.23)* | -5.74 (3.56) | -17.68 (8.91)* | -2.84 (4.90) |
| Week 53 in 2020 | | -11.55 (4.21)** | -4.93 (3.60) | -5.27 (3.56) | -16.34 (7.32)* | -9.61 (3.61)** | -26.41 (9.03)** | 0.59 (4.97) |
| Week 51 in 2021 | | 0.41 (5.12) | -0.35 (4.38) | -1.98 (4.33) | -4.67 (8.91) | -3.16 (4.40) | 1.44 (10.99) | -8.94 (6.05) |
| Week 52 in 2021 | | -4.20 (5.59) | -4.04 (4.77) | -9.74 (4.72)* | -26.39 (9.71)** | -6.79 (4.79) | -24.61 (11.98)* | -27.39 (6.59)*** |
| **R^2^** | | 0.43 | 0.35 | 0.45 | 0.67 | 0.71 | 0.78 | 0.23 |
| **Adjusted R^2^** | | 0.34 | 0.25 | 0.37 | 0.62 | 0.70 | 0.74 | 0.12 |
| **Residual Std. Error**^b^ | | 3.92 | 3.34 | 3.31 | 6.81 | 3.36 | 8.40 | 4.62 |
| **F Statistic**^c^ | | 5.09*** | 3.65*** | 5.64*** | 13.99*** | 16.8*** | 23.55*** | 2.09** |

SE, standard error; ^b:^ The degree of freedom of residual standard error was 136; ^c:^ Two degree of freedoms of F statistic were 20 and 136.

*P* value: *** <0.001, ** <0.01, * <0.05

Table S4. The interrupted time-series model of the rate of prescription for psychotropic drugs and two tracer drugs in community-dwelling older people with dementia in different phases of the COVID-19 pandemic in the southern region of the Netherlands

| **RNFM**  **Southern Region** | | **Antipsychotics** | **Anxiolytics** | **Hypnotics/Sedatives** | **Antidepressants** | **Anti-dementia Drugs** | **Statins** | **Opioids** |
| --- | --- | --- | --- | --- | --- | --- | --- | --- |
| Pre-pandemic | Intercept (SE) | 20.45 (1.22)*** | 26.13 (1.19)*** | 25.65 (1.14)*** | 53.74 (1.83)*** | 17.27 (1.45)*** | 94.81 (2.16)*** | 30.96 (1.57)*** |
|  | Time (SE) | 0.14 (0.04)*** | 0.05 (0.03) | -0.07 (0.03)* | 0.01 (0.05) | -0.02 (0.04) | -0.01 (0.06) | -0.10 (0.05)* |
| Phase 1 | Intercept change (SE) | -1.61 (2.80) | -4.42 (2.73) | 3.26 (2.61) | -8.92 (4.19)* | -3.83 (3.33) | -3.14 (4.96) | -2.41 (3.61) |
|  | Slope change (SE) | -0.03 (0.30) | -0.21 (0.29) | 0.15 (0.28) | 0.75 (0.45) | 0.37 (0.36) | 0.42 (0.53) | 0.47 (0.39) |
| Phase 2 | Intercept change (SE) | -6.89 (2.75)* | -9.92 (2.68)*** | 1.75 (2.57) | -3.70 (4.12) | 1.16 (3.28) | -5.61 (4.88) | -0.27 (3.55) |
|  | Slope change (SE) | -0.08 (0.21) | 0.23 (0.20) | -0.14 (0.19) | -0.07 (0.31) | -0.19 (0.25) | -0.11 (0.37) | -0.31 (0.27) |
| Phase 3 | Intercept change (SE) | -7.98 (3.27)* | -8.35 (3.19)** | 3.71 (3.05) | -8.62 (4.89) | -0.57 (3.89) | -23.15 (5.79)*** | -8.42 (4.22)* |
|  | Slope change (SE) | -0.36 (0.26) | 0.22 (0.25) | -0.06 (0.24) | 0.15 (0.39) | -0.27 (0.31) | 1.67 (0.46)*** | 1.27 (0.34)*** |
| Phase 4 | Intercept change (SE) | -7.77 (3.85)* | -7.39 (3.75) | 4.51 (3.59) | -7.45 (5.76) | -3.80 (4.58) | 10.33 (6.82) | 8.51 (4.97) |
|  | Slope change (SE) | -0.73 (0.33)* | -0.04 (0.33) | 0.00 (0.31) | 0.70 (0.50) | 0.15 (0.40) | 0.11 (0.59) | 0.03 (0.43) |
| Phase 5 | Intercept change (SE) | -11.99 (3.71)** | -8.00 (3.61)* | 9.76 (3.45)** | 1.19 (5.55) | 2.22 (4.41) | 6.41 (6.56) | 15.67 (4.78)** |
|  | Slope change (SE) | -0.19 (0.12) | -0.02 (0.11) | -0.12 (0.11) | -0.22 (0.17) | -0.16 (0.14) | 0.44 (0.21)* | -0.35 (0.15)* |
| Phase 6 | Intercept change (SE) | -19.92 (5.65)*** | -11.34 (5.51)* | 3.45 (5.26) | -11.75 (8.46) | -2.66 (6.72) | 0.52 (10.01) | 10.04 (7.29) |
|  | Slope change (SE) | 1.24 (0.85) | 0.28 (0.83) | 0.45 (0.79) | -0.02 (1.27) | -0.18 (1.01) | 0.66 (1.50) | 0.57 (1.09) |
| Week 1 in 2019 | | -10.10 (4.63)* | -9.69 (4.52)* | -13.58 (4.32)** | -20.77 (6.94)** | -6.76 (5.51) | -30.33 (8.21)*** | -15.86 (5.98)** |
| Week 52 in 2019 | | -9.75 (4.58)* | -9.64 (4.47)* | -5.34 (4.27) | -14.70 (6.86)* | -10.88 (5.45)* | -7.89 (8.12) | -2.49 (5.91) |
| Week 1 in 2020 | | -1.29 (4.58) | -5.41 (4.47) | -4.11 (4.28) | -0.16 (6.87) | 7.15 (5.46) | -10.60 (8.13) | -5.15 (5.92) |
| Week 52 in 2020 | | 9.22 (4.75) | 0.06 (4.63) | -3.71 (4.43) | -8.14 (7.12) | -0.14 (5.65) | -13.13 (8.42) | -11.71 (6.13) |
| Week 53 in 2020 | | -3.35 (4.82) | -5.80 (4.70) | 3.78 (4.49) | -9.46 (7.21) | 4.58 (5.73) | -30.15 (8.53)*** | -2.64 (6.22) |
| Week 51 in 2021 | | -3.82 (5.87) | -2.49 (5.72) | -1.14 (5.46) | -0.03 (8.78) | -1.14 (6.98) | -8.44 (10.39) | 3.22 (7.57) |
| Week 52 in 2021 | | -21.12 (6.39)** | -1.32 (6.23) | -4.07 (5.95) | -15.83 (9.57) | -0.89 (7.60) | -32.63 (11.32)** | -6.42 (8.25) |
| **R^2^** | | 0.30 | 0.23 | 0.25 | 0.32 | 0.17 | 0.58 | 0.38 |
| **Adjusted R^2^** | | 0.20 | 0.12 | 0.14 | 0.22 | 0.05 | 0.52 | 0.28 |
| **Residual Std. Error**^b^ | | 4.48 | 4.37 | 4.17 | 6.71 | 5.33 | 7.94 | 5.78 |
| **F Statistic**^c^ | | 2.90*** | 2.06** | 2.29** | 3.16*** | 1.43 | 9.47*** | 4.09*** |

SE, standard error; ^b:^ The degree of freedom of residual standard error was 136; ^c:^ Two degree of freedoms of F statistic were 20 and 136.

*P* value: *** <0.001, ** <0.01, * <0.05

Table S5. The interrupted time-series model of the rate of prescription for psychotropic drugs and two tracer drugs in community-dwelling older people with dementia in different phases of the COVID-19 pandemic in the northern region of the Netherlands, adjusted for quarter seasonality

| **AHON**  **Northern Region** | | **Antipsychotics** | **Anxiolytics** | **Hypnotics/Sedatives** | **Antidepressants** | **Anti-dementia Drugs** | **Statins** | **Opioids** |
| --- | --- | --- | --- | --- | --- | --- | --- | --- |
| Pre-pandemic | Intercept (SE) | 43.51 (1.24)*** | 26.04 (0.00)*** | 31.85 (1.04)*** | 110.11 (2.07)*** | 37.54 (1.06)*** | 151.96 (2.69)*** | 28.90 (1.39)*** |
|  | Time (SE) | 0.04 (0.04) | -0.06 (0.03)* | -0.01 (0.03) | -0.42 (0.06)*** | -0.14 (0.03)*** | -0.53 (0.08)*** | -0.01 (0.04) |
| Phase 1 | Intercept change (SE) | -4.71 (2.66) | -2.27 (2.30) | -3.01 (2.23) | -0.83 (4.42) | -2.29 (2.27) | -2.10 (5.75) | 0.13 (2.99) |
|  | Slope change (SE) | 0.49 (0.29) | 0.59 (0.25)* | 0.03 (0.24) | 0.38 (0.48) | 0.21 (0.25) | -0.08 (0.62) | -0.15 (0.32) |
| Phase 2 | Intercept change (SE) | -6.12 (2.75)* | 4.06 (2.37) | -3.26 (2.30) | 3.69 (4.56) | -3.56 (2.34) | -6.00 (5.93) | -2.32 (3.08) |
|  | Slope change (SE) | -0.22 (0.19) | 0.05 (0.16) | -0.08 (0.16) | 0.50 (0.31) | 0.09 (0.16) | 0.72 (0.41) | 0.13 (0.21) |
| Phase 3 | Intercept change (SE) | -7.45 (2.91)* | 3.91 (2.51) | -4.96 (2.44)* | 12.15 (4.83)* | -3.31 (2.48) | 8.92 (6.29) | -0.85 (3.27) |
|  | Slope change (SE) | 0.06 (0.25) | 0.00 (0.22) | 0.02 (0.21) | 0.86 (0.42)* | 0.38 (0.22) | 0.77 (0.55) | -0.03 (0.28) |
| Phase 4 | Intercept change (SE) | -6.17 (3.82) | 6.34 (3.30)* | 0.01 (3.20) | 25.31 (6.35)*** | 1.67 (3.25) | 23.18 (8.26)** | 0.57 (4.29) |
|  | Slope change (SE) | -0.18 (0.31) | 0.32 (0.27) | -0.17 (0.26) | 0.62 (0.51) | 0.29 (0.26) | 0.40 (0.67) | 0.02 (0.35) |
| Phase 5 | Intercept change (SE) | -6.49 (4.06)* | 7.67 (3.50)* | -4.13 (3.40) | 28.50 (6.74)*** | 3.50 (3.45) | 24.30 (8.76)** | 0.97 (4.55) |
|  | Slope change (SE) | -0.19 (0.11) | -0.14 (0.10) | -0.01 (0.09) | 0.42 (0.19)* | 0.22 (0.10)* | 0.35 (0.24) | -0.04 (0.13) |
| Phase 6 | Intercept change (SE) | -5.37 (5.06) | 5.88 (4.36) | -1.74 (4.24) | 37.49 (8.40)*** | 5.91 (4.30) | 34.61 (10.92)** | -5.32 (5.67) |
|  | Slope change (SE) | -0.34 (0.74) | -0.42 (0.64) | 0.10 (0.62) | 1.27 (1.23) | 0.97 (0.63) | 0.82 (1.60) | 1.80 (0.83)* |
| Week 1 in 2019 | | -13.45 (4.09)** | -11.64 (3.53)** | -14.63 (3.43)*** | -48.04 (6.80)*** | -23.06 (3.48)*** | -60.39 (8.84)*** | -10.98 (4.59)* |
| Week 52 in 2019 | | -0.41 (4.05) | -3.08 (3.50) | -9.19 (3.39)** | -7.05 (6.72) | -6.74 (3.44) | -19.43 (8.74)* | -0.04 (4.54) |
| Week 1 in 2020 | | -18.44 (4.10)*** | -7.70 (3.54)* | -6.91 (3.44)* | -35.06 (6.82)*** | -15.08 (3.49)*** | -49.69 (8.87)*** | -7.03 (4.61) |
| Week 52 in 2020 | | -6.23 (4.20) | -5.43 (3.63) | -7.30 (3.52)* | -12.69 (6.98) | -5.50 (3.58) | -15.79 (9.08) | -4.74 (4.72) |
| Week 53 in 2020 | | -10.50 (4.28)* | -4.71 (3.69) | -6.63 (3.58) | -12.92 (7.10) | -9.34 (3.64)* | -24.26 (9.24)** | -1.58 (4.80) |
| Week 51 in 2021 | | 0.41 (5.11) | -0.35 (4.41) | -1.98 (4.29) | -4.67 (8.49) | -3.16 (4.35) | 1.44 (11.05) | -8.94 (5.74) |
| Week 52 in 2021 | | -4.20 (5.57) | -4.04 (4.81) | -9.74 (4.67)* | -26.39 (9.26)** | -6.79 (4.74) | -24.61 (12.04)* | -27.39 (6.25)*** |
| Quarter 2 (April, May, June) | | -1.77 (1.20) | -1.03 (1.03) | 1.85 (1.01) | -3.83 (1.99) | -2.16 (1.02)* | -1.90 (2.59) | 5.34 (1.35)*** |
| Quarter 3 (July, August, Sep) | | -0.67 (1.22) | -0.56 (1.05) | 1.98 (1.02) | -8.26 (2.03)*** | -1.88 (1.04) | -1.86 (2.63) | 2.75 (1.37)* |
| Quarter 4 (Oct, Nov, Dec) | | -1.84 (1.41) ** | -0.40 (1.22) | 2.39 (1.18)* | -6.00 (2.34)* | -0.48 (1.20) | -3.77 (3.05) | 3.82 (1.58)* |
| **R^2^** | | 0.44 | 0.35 | 0.48 | 0.71 | 0.72 | 0.78 | 0.33 |
| **Adjusted R^2^** | | 0.35 | 0.24 | 0.39 | 0.66 | 0.68 | 0.74 | 0.21 |
| **Residual Std. Error**^b^ | | 3.91 | 3.34 | 3.27 | 6.49 | 3.32 | 8.44 | 4.38 |
| **F Statistic**^c^ | | 4.61*** | 3.17 *** | 5.27 *** | 14.12*** | 15.16*** | 20.36*** | 2.80 ** |
| Compare with regular model | |  |  |  |  |  |  |  |
| **Sum of squares** | | 55.95 | 11.32 | 64.03 | 706.02 | 63.59 | 122.08 | 347.89 |
| **F Statistic** | | 1.22 | 0.33 | 1.99 | 5.59** | 1.92 | 0.57 | 6.04*** |

SE, standard error; ^b:^ The degree of freedom of residual standard error was 133; ^c:^ Two degree of freedoms of F statistic were 23 and 133.

*P* value: *** <0.001, ** <0.01, * <0.05

Table S6. The interrupted time-series model of the rate of prescription for psychotropic drugs and two tracer drugs in community-dwelling older people with dementia in different phases of the COVID-19 pandemic in the southern region of the Netherlands, adjusted for quarter seasonality

| **RNFM**  **Southern Region** | | **Antipsychotics** | **Anxiolytics** | **Hypnotics/Sedatives** | **Antidepressants** | **Anti-dementia Drugs** | **Statins** | **Opioids** |
| --- | --- | --- | --- | --- | --- | --- | --- | --- |
| Pre-pandemic | Intercept (SE) | 19.51 (1.43)*** | 25.42 (1.36)*** | 24.56 (1.33)*** | 52.04 (2.10)*** | 16.69 (1.70)*** | 97.57 (2.49)*** | 27.78 (1.78)*** |
|  | Time (SE) | 0.14 (0.04)*** | 0.01 (0.04) | -0.07 (0.04)* | -0.04 (0.06) | -0.04 (0.05) | -0.04 (0.07) | -0.07 (0.05)* |
| Phase 1 | Intercept change (SE) | -0.50 (3.05) | -0.92 (2.91) | 4.20 (2.85) | -3.92 (4.49) | -1.78 (3.65) | -4.72 (5.33) | -0.48 (3.81) |
|  | Slope change (SE) | -0.16 (0.33) | -0.31 (0.32) | -0.04 (0.31) | 0.46 (0.49) | 0.28 (0.40) | 0.84 (0.58) | -0.15 (0.41) |
| Phase 2 | Intercept change (SE) | -7.67 (3.15)* | -7.83 (3.00)* | 0.48 (2.94) | -2.10 (4.63) | 2.05 (3.76) | -1.60 (5.50) | -4.84 (3.93) |
|  | Slope change (SE) | -0.10 (0.22) | 0.14 (0.21) | -0.10 (0.20) | -0.12 (0.32) | -0.23 (0.26) | -0.12 (0.38) | -0.13 (0.27) |
| Phase 3 | Intercept change (SE) | -7.83 (3.34)* | -9.29 (3.18)** | 3.49 (3.11) | -10.30 (4.91)* | -1.13 (3.98) | -24.01 (5.83)*** | -9.49 (4.17)* |
|  | Slope change (SE) | -0.32 (0.29) | 0.56 (0.28)* | -0.02 (0.27) | 0.61 (0.43) | -0.08 (0.35) | 1.72 (0.51)*** | 1.32 (0.36)*** |
| Phase 4 | Intercept change (SE) | -6.29 (4.38) | -1.42 (4.17) | 5.64 (4.09) | 0.79 (6.44) | -0.39 (5.23) | 9.02 (7.65) | 10.44 (5.47) |
|  | Slope change (SE) | -0.84 (0.35)* | -0.12 (0.34) | -0.16 (0.33) | 0.46 (0.52) | 0.08 (0.42) | 0.47 (0.62) | -0.49 (0.44) |
| Phase 5 | Intercept change (SE) | -12.68 (4.65)** | -3.07 (4.43) | 7.98 (4.34) | 5.74 (6.84) | 4.54 (5.55) | 12.07 (8.12) | 8.42 (5.81)** |
|  | Slope change (SE) | -0.18 (0.13) | -0.11 (0.12) | -0.08 (0.12) | -0.27 (0.19) | -0.20 (0.15) | 0.37 (0.23) | -0.17 (0.16)* |
| Phase 6 | Intercept change (SE) | -19.53 (5.80)*** | -8.36 (5.52) | 3.11 (5.41) | -8.65 (8.53) | -1.14 (6.92) | 1.27 (10.12) | 7.62 (7.24) |
|  | Slope change (SE) | 1.24 (0.85) | 0.32 (0.81) | 0.44 (0.79) | 0.03 (1.25) | -0.15 (1.01) | 0.68 (1.48) | 0.54 (1.06) |
| Week 1 in 2019 | | -9.16 (4.69) | -8.93 (4.47)* | -12.50 (4.39)** | -19.02 (6.90)** | -6.16 (5.60) | -33.07 (8.19)*** | -12.72 (5.86)* |
| Week 52 in 2019 | | -9.41 (4.64)* | -10.89 (4.42)* | -5.14 (4.33) | -16.34 (6.82)* | -11.53 (5.54)* | -9.69 (8.10) | -1.97 (5.79) |
| Week 1 in 2020 | | -0.32 (4.71) | -2.36 (4.48) | -3.32 (4.39) | 4.15 (6.92) | 8.92 (5.62) | -11.97 (8.22) | -3.60 (5.88) |
| Week 52 in 2020 | | 8.91 (4.82) | -2.05 (4.60) | -4.01 (4.50) | -11.09 (7.09) | -1.33 (5.75) | -13.32 (8.42) | -12.24 (6.02)* |
| Week 53 in 2020 | | -3.70 (4.91) | -8.22 (4.70) | 3.45 (4.58) | -12.83 (7.21) | 3.21 (5.85) | -30.37 (8.56)*** | -3.24 (6.12) |
| Week 51 in 2021 | | -3.82 (5.87) | -2.49 (5.58) | -1.14 (5.47) | -0.03 (8.62) | -1.14 (7.00) | -8.44 (10.24) | 3.22 (7.32) |
| Week 52 in 2021 | | -21.12 (6.39)** | -1.32 (6.09) | -4.07 (5.96) | -15.83 (9.40) | -0.89 (7.63) | -32.63 (11.16)** | -6.42 (7.98) |
| Quarter 2 (April, May, June) | | 1.39 (1.38) | 1.47 (1.31) | 1.91 (1.28) | 3.49 (2.02) | 1.15 (1.64) | -4.00 (2.40) | 5.97 (1.72)*** |
| Quarter 3 (July, August, Sep,) | | 2.23 (1.40) | 3.58 (1.33)** | 1.56 (1.31) | 5.04 (2.06)* | 2.17 (1.67) | -4.99 (2.44)* | 2.92 (1.75) |
| Quarter 4 (Oct, Nov, Dec) | | 0.63 (1.62) | 4.24 (1.54)** | 0.59 (1.51) | 5.91 (2.38)* | 2.40 (1.93) | 0.39 (2.82) | 1.06 (2.02) |
| **R^2^** | | 0.31 | 0.28 | 0.27 | 0.36 | 0.19 | 0.60 | 0.43 |
| **Adjusted R^2^** | | 0.20 | 0.16 | 0.14 | 0.25 | 0.05 | 0.53 | 0.33 |
| **Residual Std. Error**^b^ | | 4.48 | 4.27 | 4.18 | 6.59 | 5.35 | 7.82 | 5.59 |
| **F Statistic**^c^ | | 2.65*** | 2.30** | 2.10** | 3.20*** | 1.33 | 8.79*** | 4.34*** |
| Compare with regular model | |  |  |  |  |  |  |  |
| **Sum of squares** | | 59.39 | 174.73 | 45.23 | 351.47 | 59.30 | 434.67 | 385.58 |
| **F Statistic** | | 0.99 | 3.20* | 0.86 | 2.70* | 0.69 | 2.37 | 4.11** |

SE, standard error; ^b:^ The degree of freedom of residual standard error was 133; ^c:^ Two degree of freedoms of F statistic were 23 and 133.

*P* value: *** <0.001, ** <0.01, * <0.05


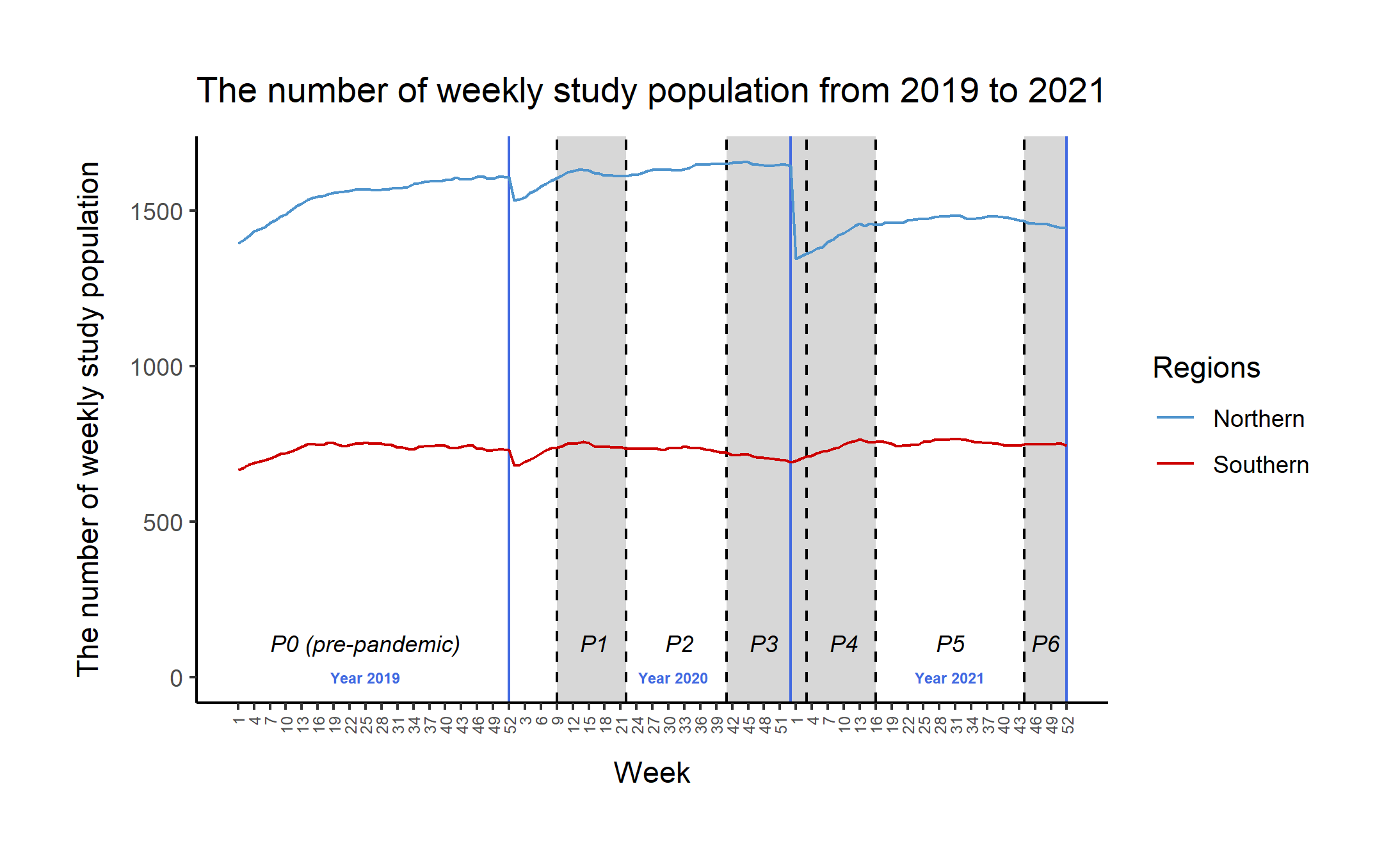


Figure S1. The number of weekly study population in the northern and southern regions from 2019 to 2021

The number of weekly study population

Note: The decrease in the amount of population in the north in 2021 was more likely to be related to the data extraction strategy. Data for 2019 and 2020 were extracted together, while data for 2021 were extracted separately.

The percentage of different types of consultations

**(a) Northern region**


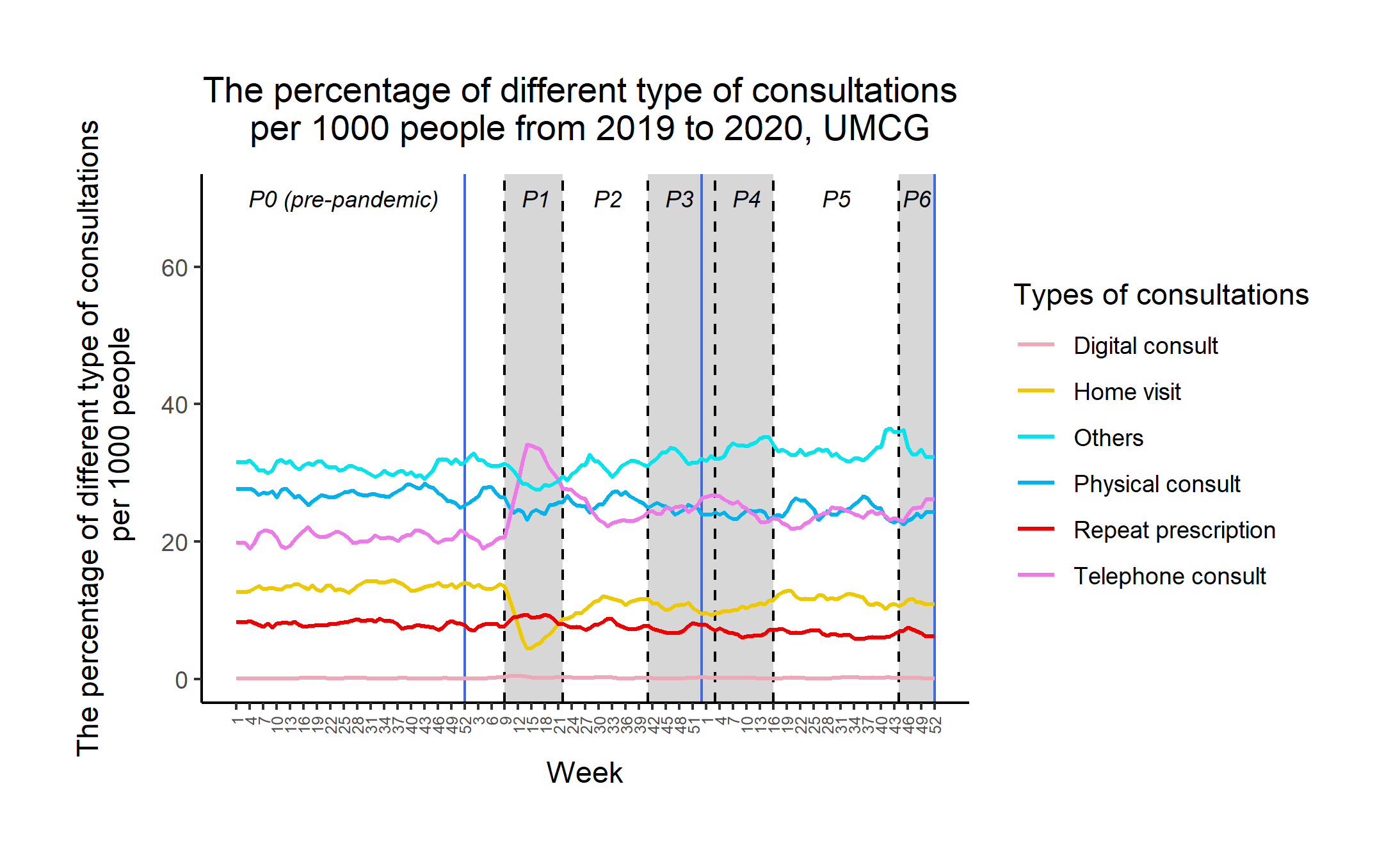


Figure S2. The percentage of different types of consultations per week from 2019 to 2021

The percentage of different types of consultations

**(b) Southern region**


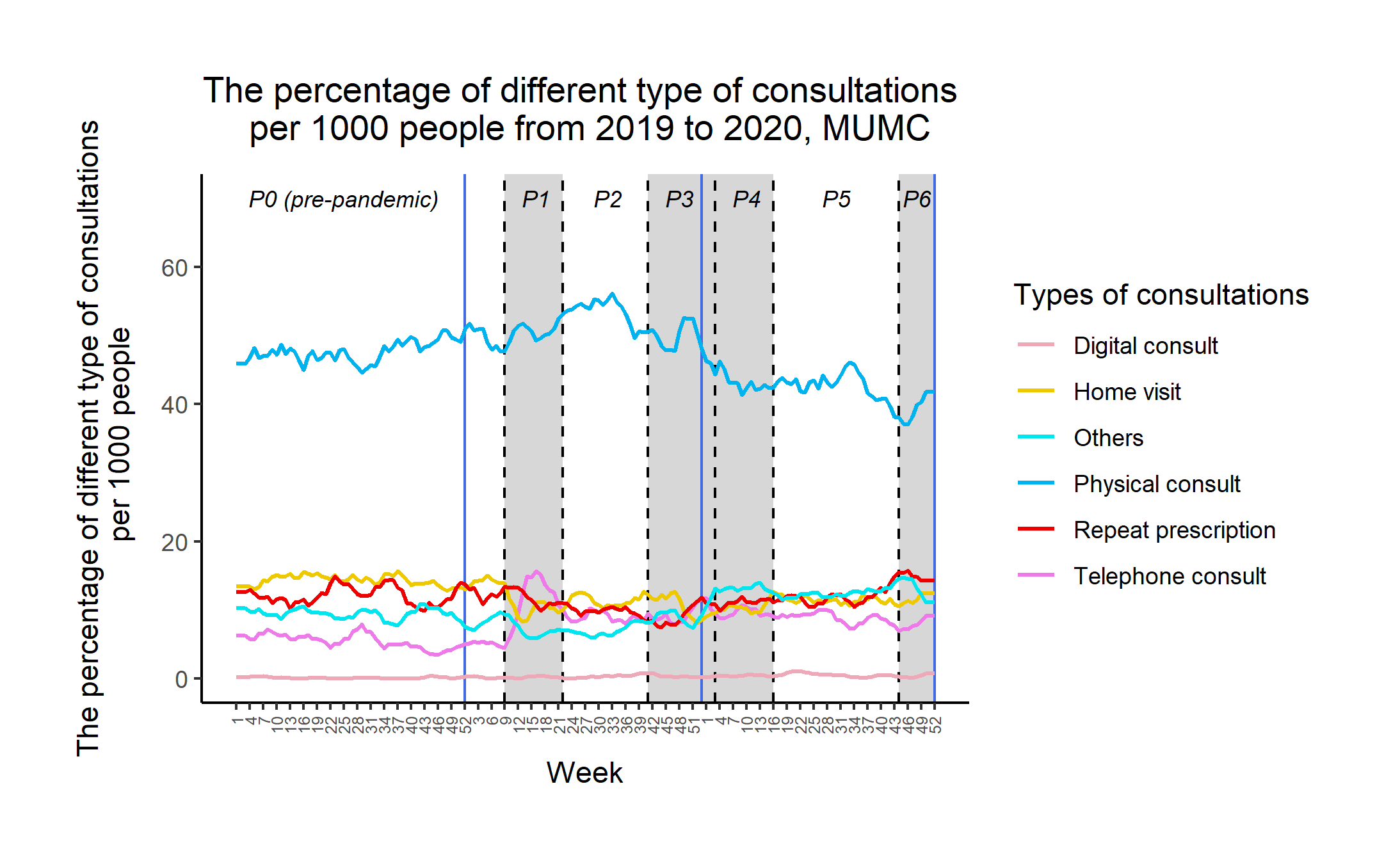


The number of consultations per 1000 people

Figure S3. The rate of weekly general practice consultations per 1000 community-dwelling older people with dementia from 2019 to 2021


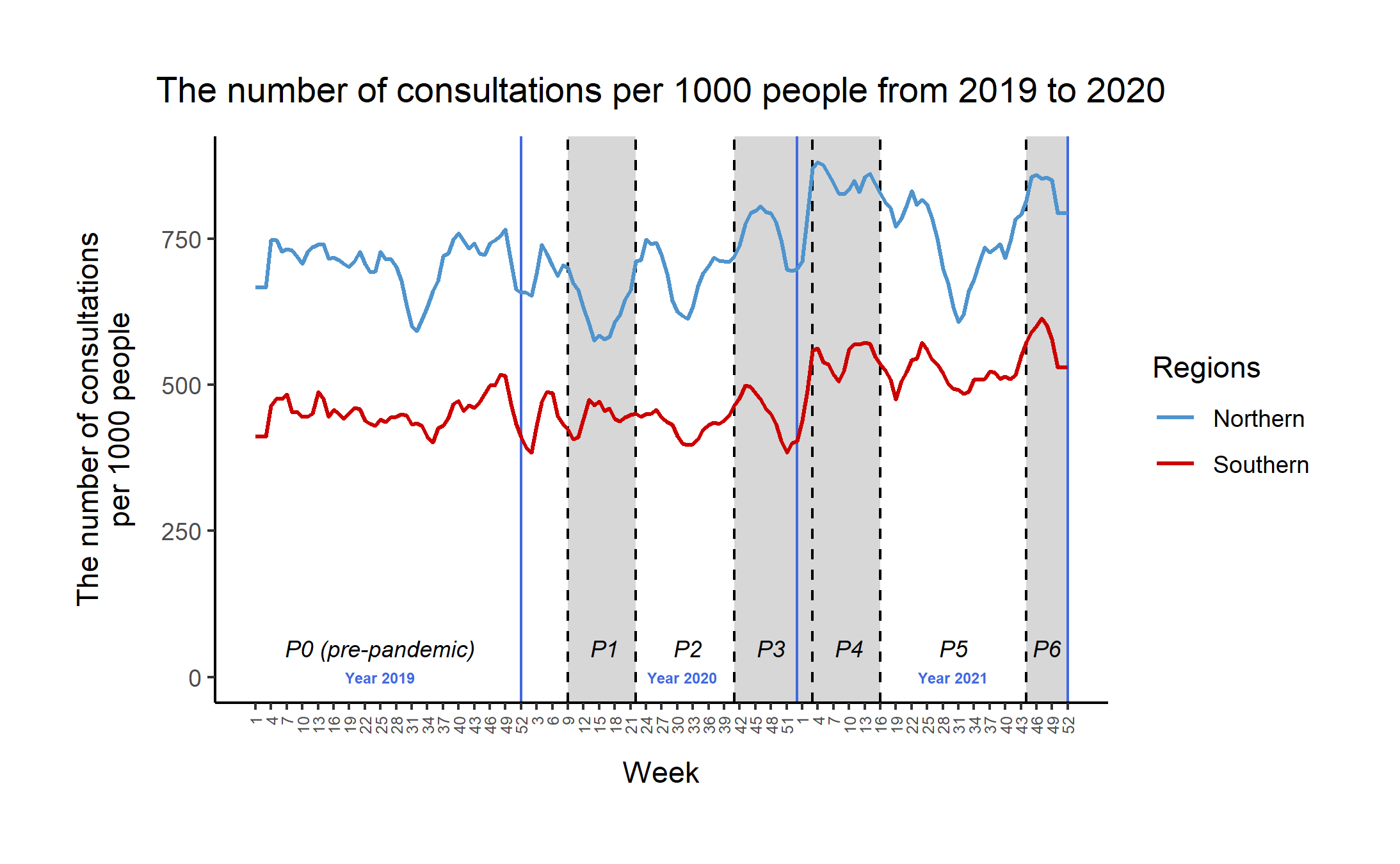


The absolute change in

the number of weekly consultations

Figure S4. The absolute change in the rate of weekly general practice consultations per 1000 community-dwelling older people with dementia, compared with corresponding weeks in 2019


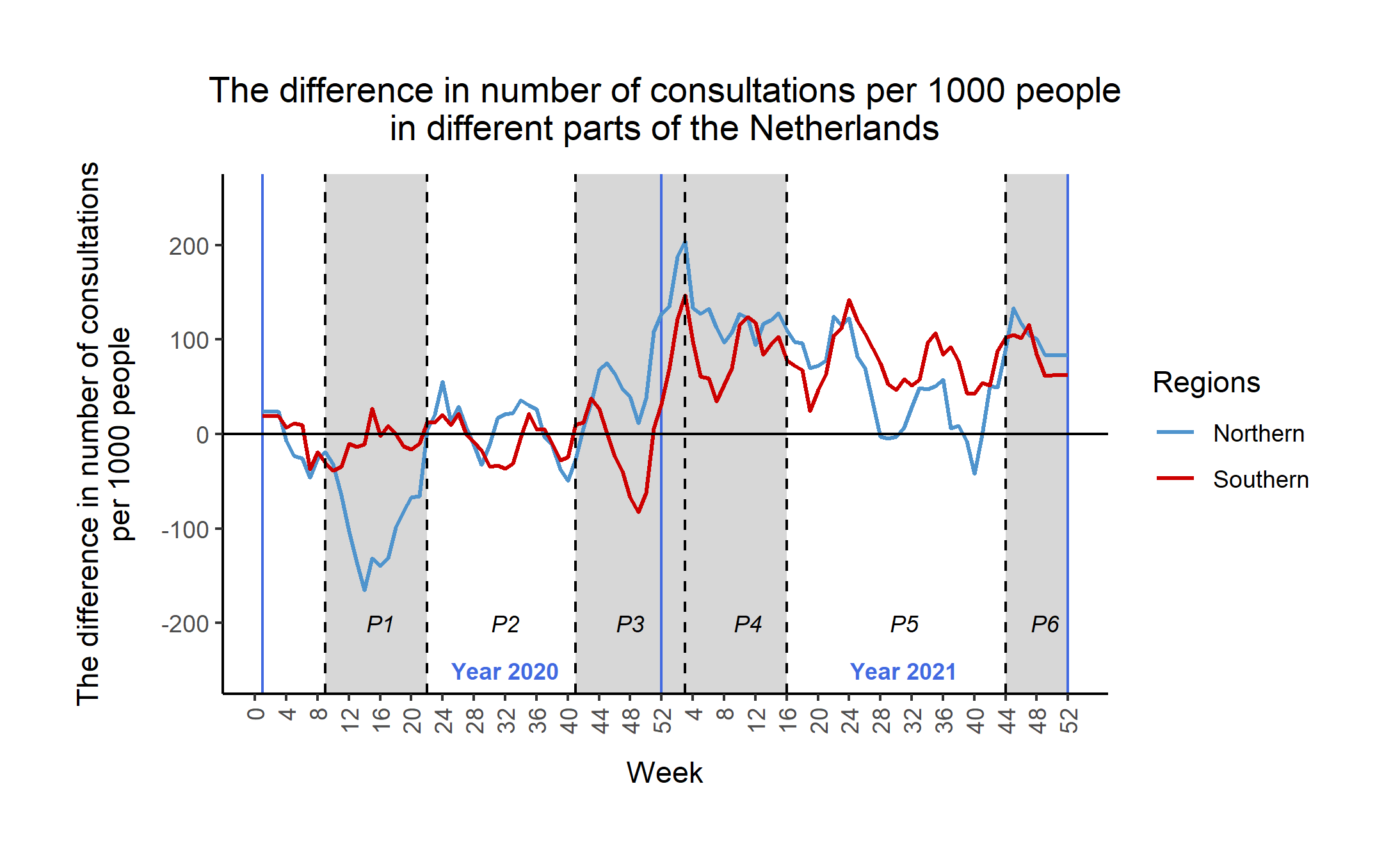


Change in the number

(a) Antipsychotics


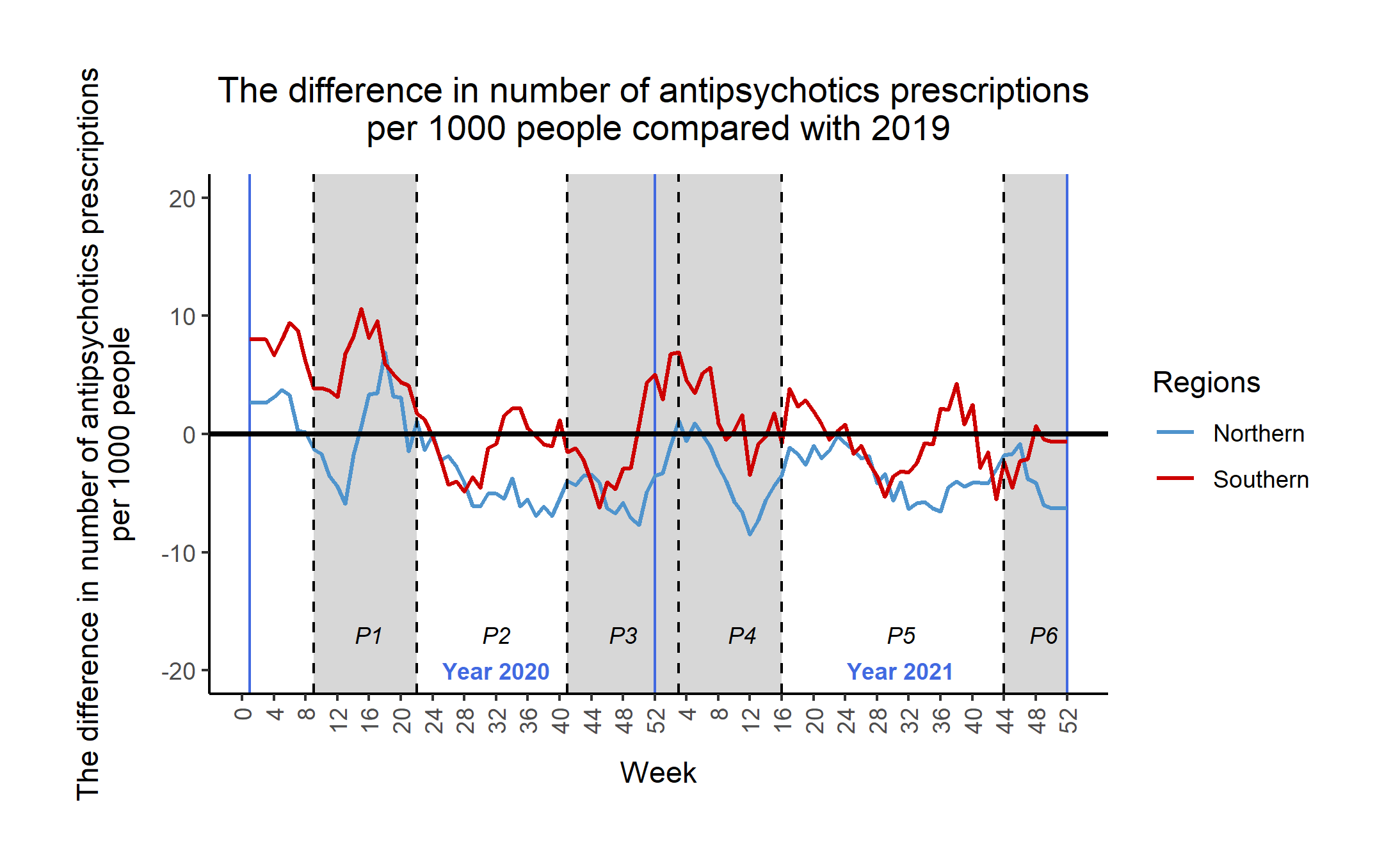


Figure S5. The absolute change in the rate of weekly prescriptions per 1000 community-dwelling older people with dementia, compared with corresponding weeks in 2019

Change in the number

(b) Anxiolytics


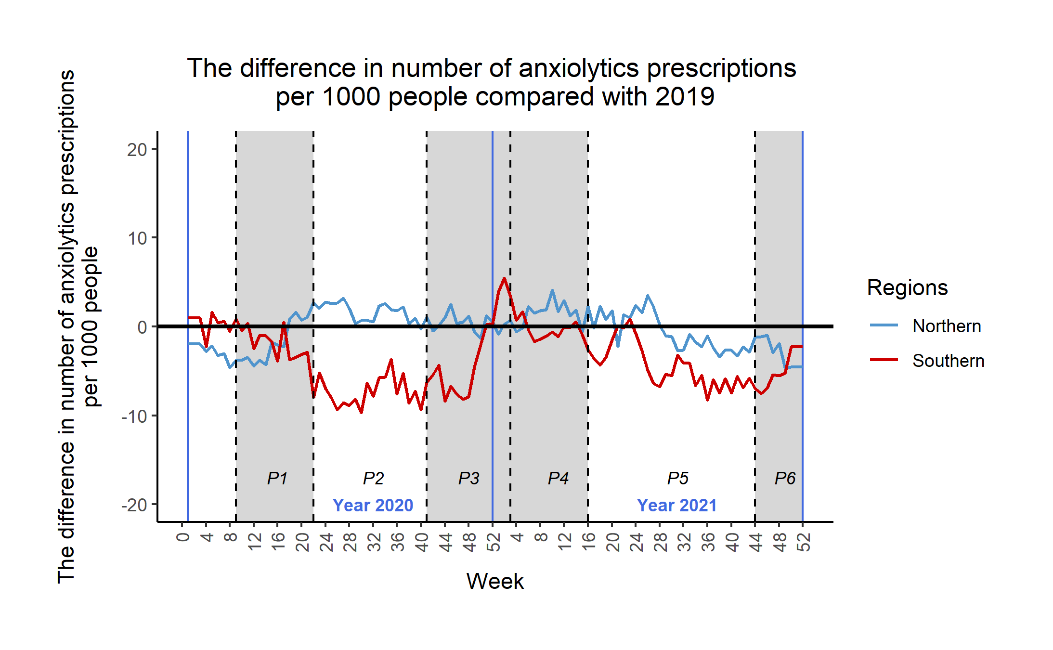


Change in the number

(d) Antidepressants


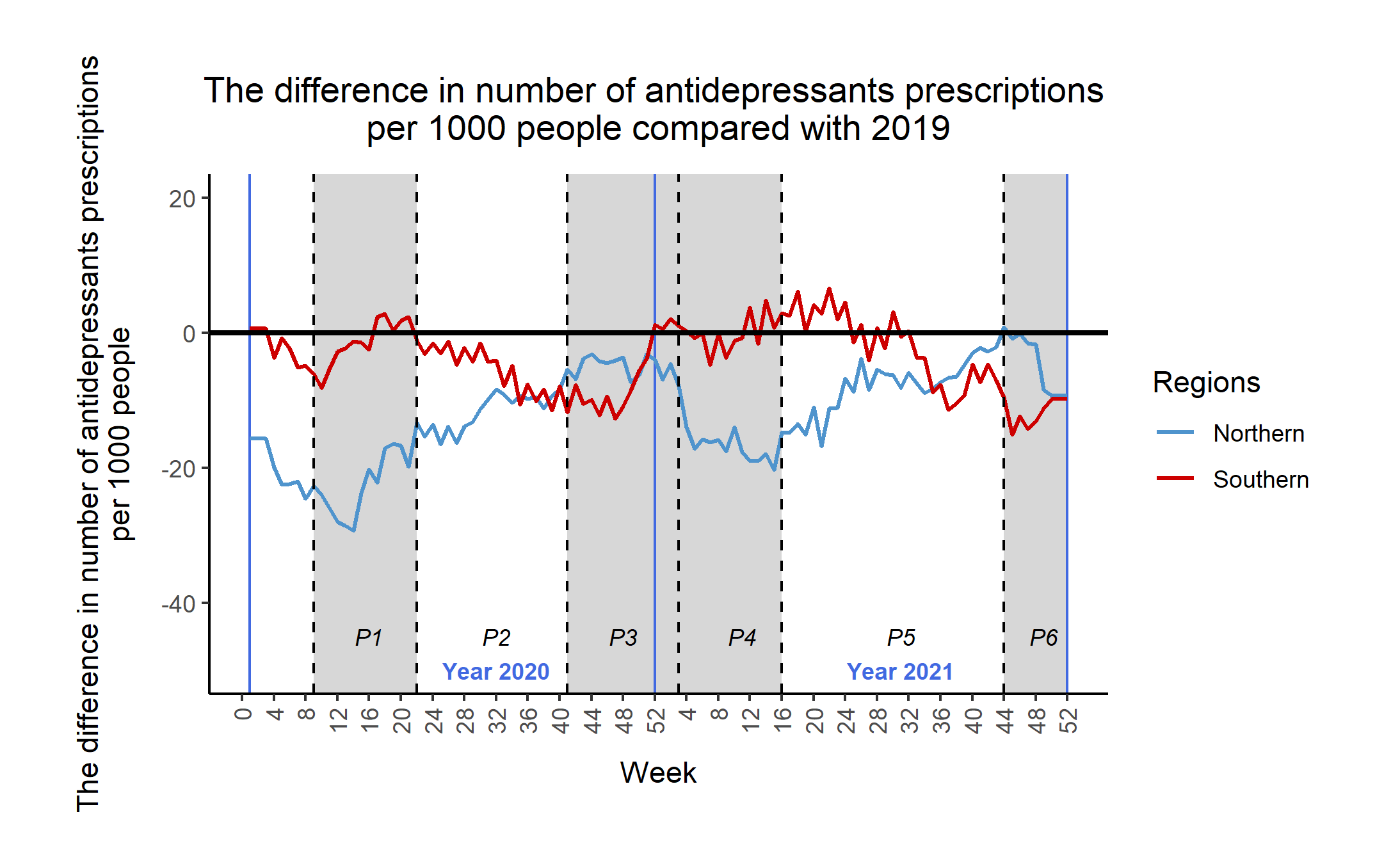


Change in the number

(c) Hypnotics/Sedatives


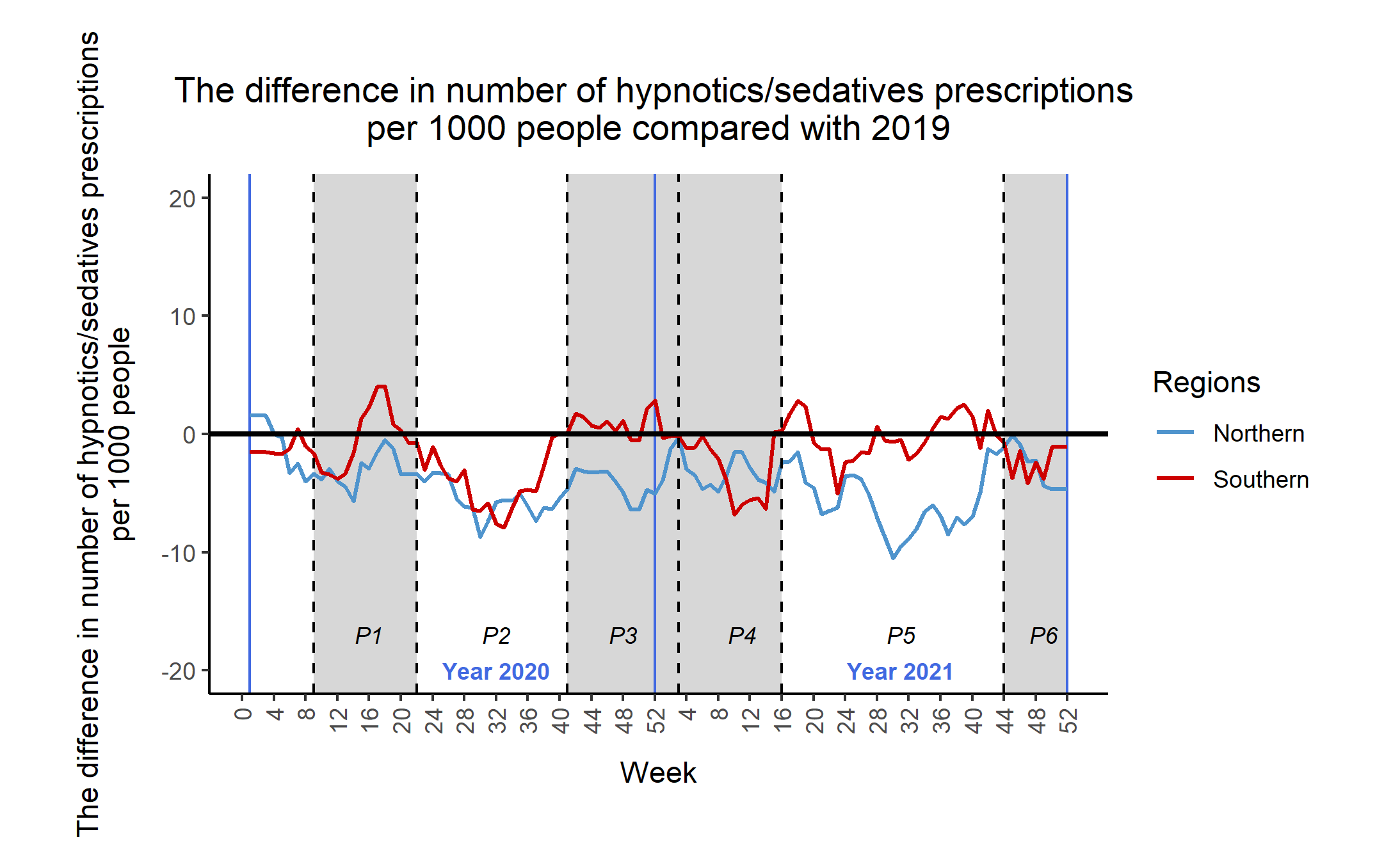


Change in the number

(e) Anti-dementia drugs


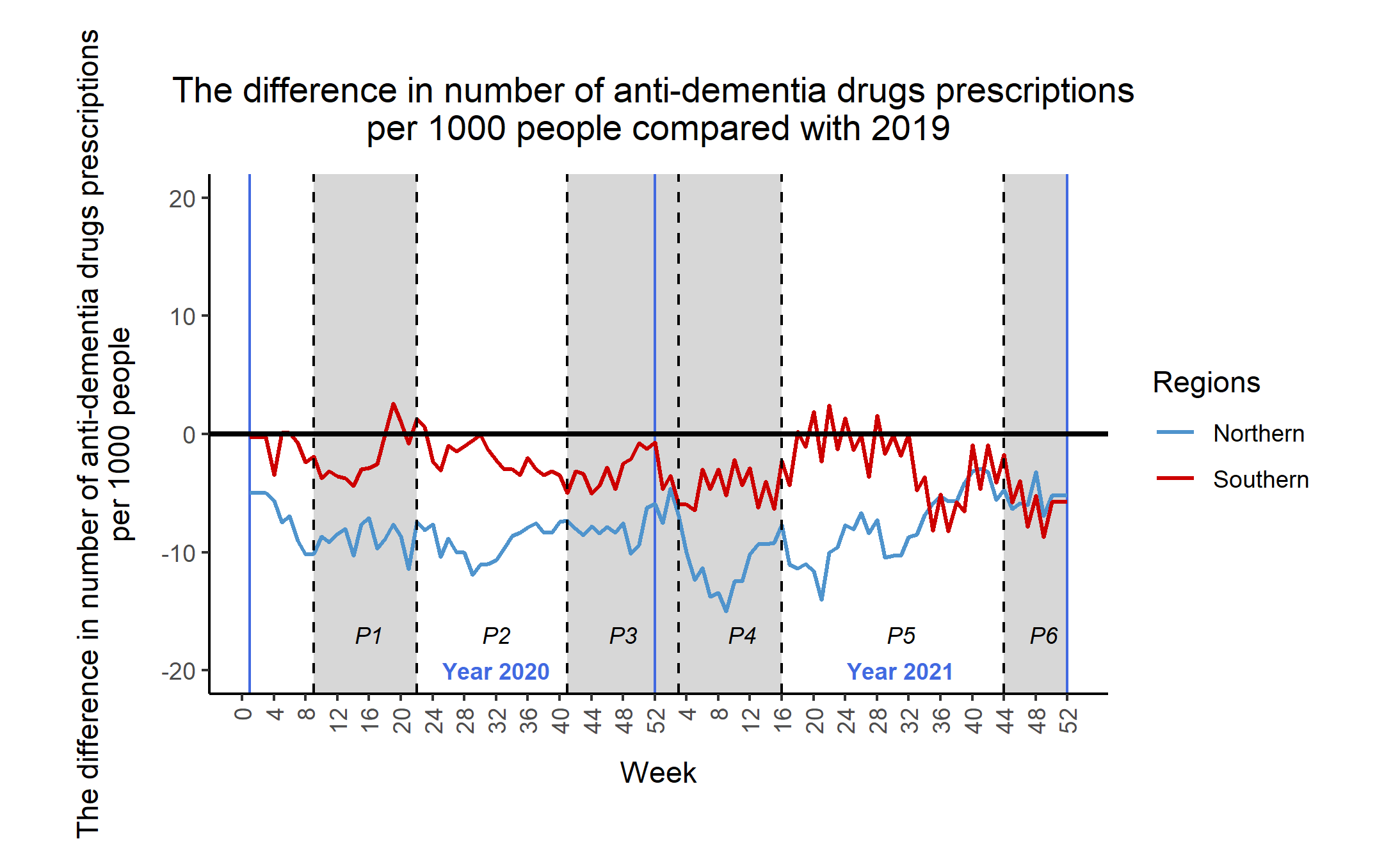


Change in the number

(f) Statins


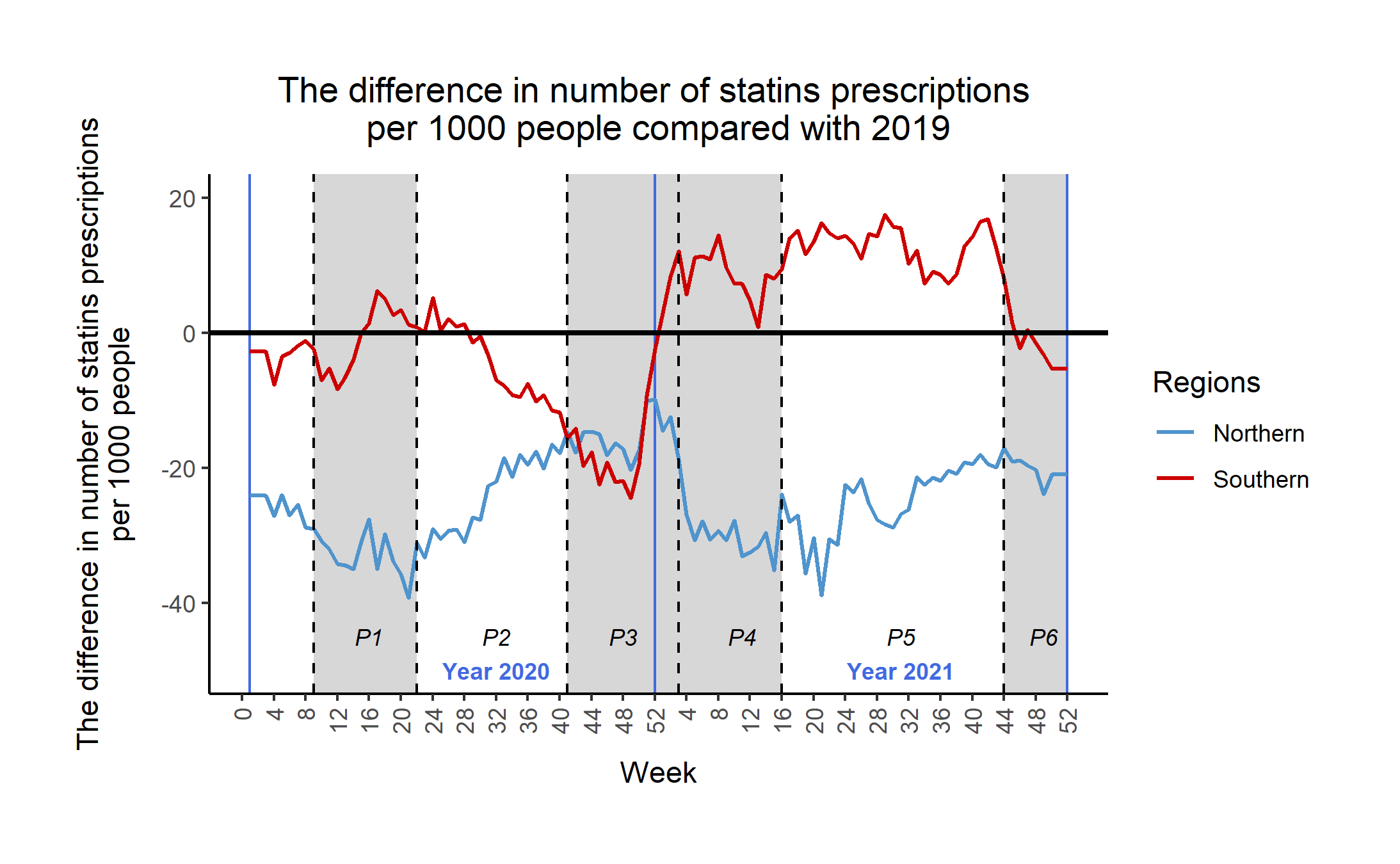


Change in the number

(g) Opioids


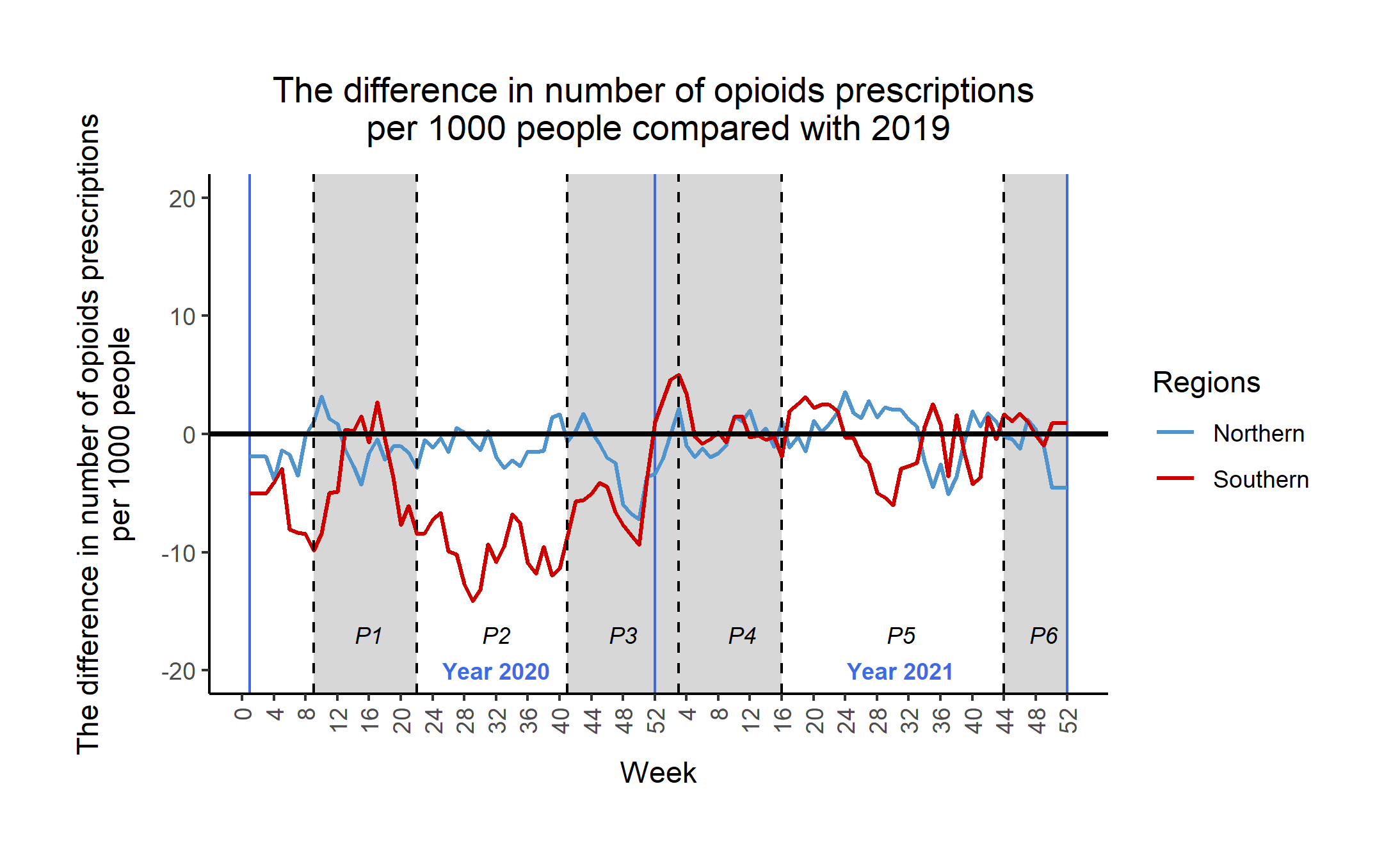

Supplement: Supplementary file 1 — Additional file 1: Table S1. Flow chart for the recruitment of the study population in different research networks each year. Table S2. The number of prescriptions for psychotropic drugs and two tracer drugs, and general practice consultations of community-dwelling older people with dementia in different research networks by year (2019-2021). Table S3. The interrupted time-series model of the rate of prescription for psychotropic drugs and two tracer drugs in community-dwelling older people with dementia in different phases of the COVID-19 pandemic in the northern region of the Netherlands. Table S4. The interrupted time-series model of the rate of prescription for psychotropic drugs and two tracer drugs in community-dwelling older people with dementia in different phases of the COVID-19 pandemic in the southern region of the Netherlands. Table S5. The interrupted time-series model of the rate of prescription for psychotropic drugs and two tracer drugs in community-dwelling older people with dementia in different phases of the COVID-19 pandemic in the northern region of the Netherlands, adjusted for quarter seasonality. Table S6. The interrupted time-series model of the rate of prescription for psychotropic drugs and two tracer drugs in community-dwelling older people with dementia in different phases of the COVID-19 pandemic in the southern region of the Netherlands, adjusted for quarter seasonality. Figure S1. The number of weekly study population in the northern and southern regions from 2019 to 2021. Figure S2. The percentage of different types of consultations per week from 2019 to 2021. Figure S3. The rate of weekly general practice consultations per 1000 community-dwelling older people with dementia from 2019 to 2021. Figure S4. The absolute change in the rate of weekly general practice consultations per 1000 community-dwelling older people with dementia, compared with corresponding weeks in 2019. Figure S5. The absolute change in the rate of weekly prescript [file 12877_2024_4708_MOESM1_ESM.docx]
